# A closer look at the WHO cone bioassay: video analysis of the hidden effects of a human host on mosquito behaviour and insecticide contact

**DOI:** 10.1186/s12936-022-04232-4

**Published:** 2022-07-01

**Authors:** Angela Hughes, Agnes Matope, Mischa Emery, Keith Steen, Gregory Murray, Hilary Ranson, Philip J. McCall, Geraldine M. Foster

**Affiliations:** Department of Vector Biology, Liverpool School of Tropical Medicine, Pembroke Place, Liverpool, L3 5QA UK

**Keywords:** Mosquito, Vector, Behaviour, Anopheles, ITN, Pyrethroid, Insecticide, Control, Bednet, Bioassay, Malaria

## Abstract

**Background:**

The WHO cone test is one of three tests currently used to evaluate the efficacy of insecticide-treated bed nets (ITNs). It generates two test outputs, knockdown and 24-h mortality, both indicative of immediate toxicity but that reveal little about the nature of mosquito and ITN interaction or how results translate to real-world settings.

**Methods:**

A human arm held 5 mm behind the net surface acted as a host attractant during cone tests and a smartphone was used to capture mosquito behaviour in the cone. Post-exposure blood feeding and survival for nine days were recorded; ingested blood meal size was determined by measuring excreted haematin. Four strains of *Anopheles gambiae* (insecticide susceptible: Kisumu and N’gousso; insecticide resistant: Banfora and VK7) were tested with and without the host attractant using untreated, Permanet 2.0 and Olyset nets. Video recordings were scan sampled every five seconds to record mosquito positions on either the net, in flight or in contact with the cone. Generalized estimating equations were used to analyse all data except survival within nine days which was analysed using Weighted Cox Regression.

**Results:**

Net contact was the most frequently recorded behaviour in all *Anopheles* spp*.* strains on all nets. Adding the human host as attractant triggered excitatory behaviours: in all strains, the magnitude of net contact was significantly decreased compared to tests without a host. ITN exposure altered the observed behaviour of the two susceptible strains, which exhibited a decreased response to the host during ITN tests. The resistant strains did not alter their behaviour during ITN tests. Significantly less net contact was observed during Olyset Net tests compared to Permanet 2.0. The host presence affected survival after exposure: Banfora and VK7 mosquitoes exposed to Permanet 2.0 with a host lived longer compared to tests performed without a host. However, mosquitoes that blood-fed and survived long enough to digest the blood meal did not exhibit significantly reduced longevity regardless of the presence of the host attractant.

**Conclusions:**

Simple modifications to the WHO cone test and extension of post-test monitoring beyond the current 24 h enable detailed behavioural characterizations of individual ITNs to be compiled. The effects observed from testing with a host and including blood feeding suggest that more representative estimates of true of ITN efficacy are gained with these modifications than when using the current testing protocol.

**Supplementary Information:**

The online version contains supplementary material available at 10.1186/s12936-022-04232-4.

## Background

Insecticide-treated nets (ITNs) are a fundamental tool in the continuing drive for sustainable malaria reduction and elimination in at-risk communities in Africa [1–3]. Widespread insecticide resistance in the major African malaria vectors *Anopheles gambiae *sensu lato (*s.l.*) and *Anopheles funestus s.l.* has reduced the entomological efficacy of ITNs; by reducing mosquito mortality, the critical community effect achieved through widespread ITN usage is diminished [4–9]. Although in some resistant mosquito populations sub-lethal insecticide doses can deliver reductions in vector longevity below that required to transmit the malaria parasite, studies conducted in highly-resistant vector populations in Burkina Faso indicate that as resistance intensity increases, such sub-lethal impacts disappear [10, 11]. In the current landscape where a plurality of resistance states exists across African Anopheline vector populations, a nuanced approach to ITN efficacy evaluations that characterizes mosquito-ITN interactions and incorporates measures of insecticide-induced impairment has the potential to deliver additional insights, especially in the advent of combination ITNs containing two or more active ingredients [12, 13].

Laboratory measurements of ITN entomological efficacy predominantly depend on results from the WHO cone test, which was introduced in the 1980s and is designed to capture the rapid toxicity effects of pyrethroid insecticides using two post-exposure endpoints: knock down (KD) at 60 min and 24 h mortality [14, 15]. The WHO cone test does not incorporate a host to which the mosquito can respond, unlike the other two procedures described in the current World Health Organization (WHO) ITN testing guidelines, the tunnel test and experimental hut trials [16]. However, the secondary tunnel test incorporates a non-human host, typically a rabbit or guinea pig, and experimental hut trials incorporate a human host but are performed after the laboratory stages of efficacy evaluations are complete [16]. Thus, the laboratory evaluations of ITN efficacy are primarily performed using a test that does not assess the efficacy of insecticide-treated materials in the presence of human prey, and for which the limited applicability of results to ‘real life’ situations has previously been noted [17].

To understand how effective an ITN might be once deployed into communities, there is an urgent need for bioassays that are able to characterize vector behavioural responses to, and downstream insecticidal effects of, ITN exposure. Such bioassays should be reasonably high-throughput, produce test outputs that capture a range of ITN modes of action, and able to be readily implemented into existing laboratory and field station set ups [18, 19]. Systems using infra-red video capture and tracking to characterize the entomological mode of action of ITNs have been previously described, but these tests are large-scale, difficult to transport and expensive, rendering them unsuitable for mass implementation into evaluation laboratories and stations [13, 20].

This report describes investigations of adaptations to the WHO cone test that allow the characterization of behavioural responses to ITNs through the incorporation of a host attractant and the addition of video capture. An extended post-exposure monitoring pipeline is implemented to assess the contribution of insecticide resistance to the lifetime impact of active ingredient (AI) exposure.

## Methods

### Mosquito colonies

All experiments were performed at the Liverpool School of Tropical Medicine (LSTM) using unfed 3–5 day old female *Anopheles gambiae s.l.* adults. Four strains of *An. gambiae s.l.* mosquitoes were used in the experiments, two of which were insecticide susceptible (Kisumu, N’gousso) and two which were insecticide resistant (Banfora, VK7).

The *An. gambiae *sensu stricto (s.s.) Kisumu (KS) colony originated in Kenya [21] and has been maintained at LSTM since 1975. *Anopheles coluzzii* N’guosso (NG) was colonized from Cameroon in 2006 [22] and is susceptible to most classes of insecticide with low level of resistance to organochlorides, DDT and Dieldrin (LSTM profiling results). The *An. coluzzii* VK7 (Vallee du Kou, village no. 7) and Banfora (BF) strains from Burkina Faso were colonized in 2014, are resistant to pyrethroids and DDT [23–25] and are maintained under six-monthly selection pressure with deltamethrin. Both insecticide resistant strains have a high frequency of 1014F *kdr* and express elevated levels of P450s known to metabolize pyrethroids [25].

### Insecticidal netting

Untreated polyester net (Bayer, Germany) was used as a baseline control. Two commercial ITNs were tested: PermaNet^®^2.0 (Vestergaard Frandsen, Switzerland, deltamethrin 1.4–1.8 g/kg, P2) and Olyset^®^ Net (Sumitomo Chemical Ltd, Japan 2% (w/w) permethrin 20 g/kg, OS). ITNs were donated by the manufacturer, aired at ambient temperature for one week, then stored at 4 °C until acclimatization at laboratory temperature 24 h prior to testing.

### Video Cone Test (VCT) apparatus and design

**Fig. 1:**
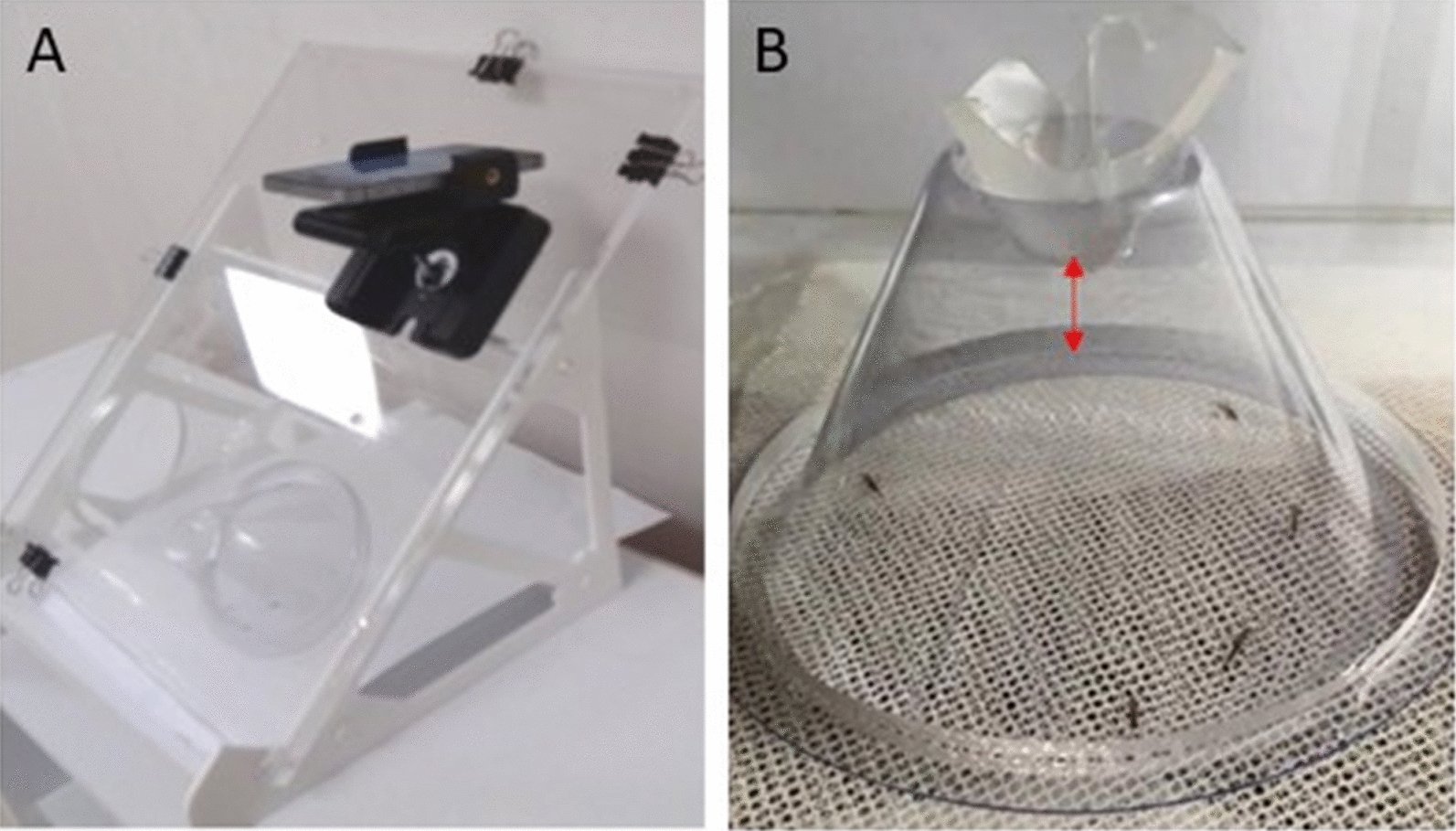
VCT apparatus and design. A bespoke stand made from fused PLA plastic filament (**A**) supports the Perspex cone board at 45°. Openings in the side of the stand allows the host arm to be placed horizontally behind the net. The smartphone is clamped to the board and positioned in front of the cone. The recording field (**B**) comprises of the entire volume of the cone and net. The presence of a gap between the lower rim and upper opening of the cone (red arrow) ensures the optimal camera angle and position

Two modifications were introduced to the WHO cone test [16]: (a) a basic smartphone (iPhone SE, release date Mar 2016) with video capture (60 frames per second (fps), 1080p HD, Apple Inc, USA); (b) a human attractant (host arm), positioned approximately 5 mm from the test netting. The smartphone was clamped to a 420 mm × 297 mm (H × W) cone board held at 45° by a bespoke fused PLA plastic filament base (237 mm × 280 mm × 266 mm (H × W × D) (Fig. 1A) and positioned such that the entire cone and net were visible in the recording field (Fig. 1B).

### Experimental protocol

All experiments were performed in a climate-controlled laboratory (27 ± 2 °C, 80 ± 8% RH) after the first hour of the scotophase using mosquitoes acclimatized to laboratory conditions. Prior to testing, mosquitoes were starved of 10% sugar solution for at least five hours.

### VCT

A minimum of 200 mosquitoes each of Kisumu, N’gousso, VK7 and Banfora strains were exposed to P2, OS and untreated nets. A total of 100 mosquitoes were exposed in the presence of the host attractant, (host present tests, HP) and 100 without (host absent tests, HA). Observed differences in mosquito behaviour between: (a) the presence and absence of the host attractant, and (b) untreated and insecticide-treated netting materials, were evaluated.

After exposure, 60-min KD, 24-h mortality and life history traits were recorded.

The number of replicates performed per day were allowed to fluctuate according to mosquito availability, to mimic routine availability in field conditions, with a minimum number of three test days per strain using untreated net and two days using each treated net to capture routine day-to-day test variation. To reduce inter-person variation, a single host was used for all host present tests.

### Video analyses

Videos were converted to Windows Media Video (WMF) format using Windows Movie Maker (©2012 Microsoft Corporation, USA) and analysed using event-logging software BORIS [26]. The positions of all five mosquitoes were categorized as either ‘net’ (in contact with the net),’cone’ (in contact with the cone) or ‘in flight’ at five-second intervals for the duration of the 180-s assay, producing 36 data points per three-minute test. These data points were used for the statistical comparisons of observed mosquito behaviours in the cone.

### Life history traits

Blood feeding at 1- and 24-h post-exposure, blood meal size and longevity were recorded. Briefly, after recording KD at 60 min, mosquitoes were given the opportunity to blood feed for 20 min on a human host arm. Mosquitoes that blood fed were transferred into individual 50 ml Falcon tubes with a lid of untreated netting to enter the monitoring pipeline. Unfed mosquitoes were offered a second blood meal 24 h post-exposure, and then transferred to individual Falcon tubes to begin monitoring. Seventy-two hours after mosquitoes were transferred to the Falcon tubes, mosquitoes were transferred into a fresh Falcon tube and the original housing was used to measure individual blood meal sizes using excreted haematin [27]. Briefly, the excreted haematin collected in individual falcon tubes was dissolved in 1 ml 1% Lithium carbonate and the absorbance at 397 nm measured in triplicate. The total concentration of haematin (µg/ml) in each sample was determined using a seven point standard curve comprising standards from 1.76 to 20 µg/ml. As the concentration of haematin is directly stoichiometrically related to haemoglobin input, which is indicative of the total volume of blood ingested, the haematin measurements were used as a measurement of ingested blood meal size [28].

Mosquitoes were maintained in individual housing with access to 10% sugar solution and mortality was recorded every 24 h until natural death, after which they were stored at 20 °C until measuring wing‐length as an index of mosquito mass [29]. The right wing of each mosquito was removed and mounted onto glass slides, and a GXCAM ECLIPSE Wi-Fi camera attached to a GX Stereo microscope (GT Vision Ltd) was used to capture an image. The length of the wing was measured from the axial vein to the distal end of the R1 vein using GXCAM software (GXCAM Ver.6.7).

### Statistical analysis

Descriptive statistics were generated using number of observations, mean, standard deviation (SD), minimum, maximum, and coefficient of variation for continuous variables results, the number and percentage of observations for categorical variables, and number and percentage of events, mean, SD, minimum, maximum, median survival and its corresponding 95% confidence interval (CI) for time-to-event variables.

Within- and between-day test variability was used to evaluate VCT robustness. Variability of less than 30% within each *An. gambiae* strain in host absent tests using untreated net were considered to comply with previous measurements of variability within cone tests [30]. As the addition of a host attractant to the test was assumed to prompt behavioural changes in the test mosquitoes, and as no published precedent regarding small-scale responses to ITNs exists, an additional 10% variability in within- and between-day imprecision in host present untreated net tests were set as putative acceptability criteria for test acceptance. Any additional variability in behaviour during ITN tests was therefore assumed to be driven by the insecticidal net treatment.

A marginal logistic regression model using generalized estimating equations (GEE) was employed to analyse mosquito behaviour in the cone. The variance component covariance structure was used due to non-convergence issues of other complex covariance structures. The beta-binomial distribution and a logit link function were considered for the analysis, with the mean proportion of mosquitoes in contact with the net and flight or cone for the entire assay as an outcome. Host (absent or present), strain, treatment, location (net, flight or cone), and all the possible interactions among these variables were fitted as fixed effects and replicate as a random effect. The odds ratio (OR) together with its corresponding 95% CI of the proportion of contact between net and flight/cone were generated. Cone contact in all assays was minimal and unfluctuating; these data were combined with flight data for the statistical analyses. Comparisons were conducted comparing the tests where the host was present or absent within a net treatment (untreated, P2, OS) and within a test comparing the net treatments. Due to the multiple comparisons that were performed, the Bonferroni adjustment procedure was employed to control for the probability of making false positive findings.

For the life history traits, GEE was used to analyse the blood feeding (at 1 h or 24 h) post-exposure and blood meal volume, using the binomial distribution with a logit link function, and a normal distribution with an identity link function, respectively. Longevity within 9 days was analysed using Weighted Cox Regression to generate unbiased averaged hazard ratios (HR) together with their corresponding 95% CI since the proportional hazard assumption was violated [31]. This analysis was performed using the R Package coxphw with replicate as a cluster [32]. Treatment, wing-length measurement, time the mosquito fed post-exposure (1 h, 24 h, no feeding or dead), blood meal volume and the predicted mean proportion of mosquitoes in contact with the net were considered as fixed effects and replicate as a cluster. All comparisons were performed within strain and host (net treatment vs untreated) or treatment (host present vs absent).

All statistical tests were conducted at 5% significance level. Statistical analysis was performed using SAS software, Version 9.4 (© 2002–2012 SAS Institute Inc., Cary, NC, USA) and R 4.0.1 [33].

### Ethical approval

Ethical approval was not required for this study as all activities were considered to fall under daily routine laboratory and colony maintenance tasks. Members of staff who performed post-exposure arm feeding were registered with LSTM as mosquito colony arm feeders and had previously signed consent forms which were kept on file. Arm feeding was considered part of routine daily colony maintenance activities.

## Results

### Evaluation of VCT robustness

Between 105 and 125 (host absent) and 100 and 130 (host present) *An. gambiae* mosquitoes per strain were used in untreated net tests (Additional file 1: Table S1), with a minimum of three test days per strain (maximum seven days, Additional file 1: Table S2). Net contact passed the robustness criteria for both within- and between-day imprecision, regardless of the number of replicates performed per day (Additional file 1: Tables S1 and S2). The total test imprecision for net contact was < 30% for each strain in host absent tests (KS 24.19%; NG 17.11%; BF 14.69%; VK7 13.56%, Additional file 1: Table S1) and < 40% for each strain in host present tests (KS 22.04%; NG 30.04%; BF 29.90%; VK7 26.19%, Additional file 1: Table S1); within each test day the maximum imprecision observed was 29.43%, 23.55%, 18.37% and 15.35% for Kisumu, N’gousso, Banfora and VK7 strains in host absent tests and 26.14%, 39.64%, 32.99%, 27.08% for Kisumu, N’gousso, Banfora and VK7 strains, respectively, in host present tests (Additional file 1: Table S2). No trends in imprecision were noted when net contact results were binned by timepoint (Additional file 1: Table S3), indicating the stability of using net contact as a measure of response.

Both flight and cone contact were too variable to be used as a robust measure of mosquito response (Additional file 1: Tables S1 and S2); results and interpretations presented hereafter use net contact as the basis for evaluation.

### Host and ITN effects on *An. gambiae* strains

The most frequently occurring observed behaviour in all *An. gambiae* strains during baseline, untreated net, host absent tests was net contact (Table 1, Host absent net vs Host absent flight KS OR 18.07; 95% CI 17.16, 19.03; *P* ≤ 0.0001; NG OR 40.79; 95% CI 39.18, 42.46; *P* ≤ 0.0001; BF OR 56.43; 95% CI 54.12, 58.85; *P* ≤ 0.0001; VK7 OR 86.26; 95% CI 83.11, 89.53, *P* ≤ 0.0001). The addition of a host attractant triggered excitatory behaviours: in all four strains the magnitude of net contact decreased significantly (Table 1, Host present net vs Host present flight KS OR 16.41; 95% CI 15.83, 17.01; *P* ≤ 0.0001; NG OR 17.44; 95% CI 15.78, 19.27; *P* ≤ 0.0001; BF OR 6.25; 95% CI 5.96, 6.56; *P* ≤ 0.0001; VK7 OR 7.25; 95% CI 6.98, 7.53; *P* ≤ 0.0001; full multivariable analysis in Additional file 1: Table S4). Notably, the two resistant strains spent approximately three times longer in contact with the net when the host was absent compared to present, indicating a higher excitatory response to the host presence than shown by the two susceptible strains (Table 1, Host absent net vs host present net KS OR 1.05; 95% CI 1.00, 1.10; *P* = 0.0256; NG OR 1.53; 95% CI 1.42, 1.65; *P* ≤ 0.0001; BF OR 3.01; 95% CI 2.87, 3.14; *P* ≤ 0.0001; VK7 OR 3.45; 95% CI 3.32, 3.58; *P* ≤ 0.0001).

**Table 1:** Host-Location comparisons within *An. gambiae s.l.* strain and net treatment from a Beta-binomial Distribution model

Net	Comparison	Odds ratio (95% bonferroni adjusted confidence interval); bonferroni adjusted P-value			
		Kisumu (IS)	N’gousso (IS)	Banfora (IR)	VK7 (IR)
UT	Host absent net *vs* host absent flight/cone	18.07 (17.16, 19.03); < 0.0001*	40.79 (39.18, 42.46); < 0.0001*	56.43 (54.12, 58.85); < 0.0001*	86.26 (83.11, 89.53); < 0.0001*
	Host present net *vs* host present flight/cone	16.41 (15.83, 17.01); < 0.0001*	17.44 (15.78, 19.27); < 0.0001*	6.25 (5.96, 6.56); < 0.0001*	7.25 (6.98, 7.53); < 0.0001*
	Host absent net *vs* host present net	1.05 (1.00, 1.10); 0.0256*	1.53 (1.42, 1.65); < 0.0001*	3.01 (2.87, 3.14); < 0.0001*	3.45 (3.32, 3.58); < 0.0001*
P2	Host absent net *vs* host absent flight/cone	18.22 (17.66, 18.81); < 0.0001*	10.44 (10.01, 10.89); < 0.0001*	47.85 (46.29, 49.46); < 0.0001*	10.02 (8.92, 11.26); < 0.0001*
	Host present net *vs* host present flight/cone	18.69 (17.63, 19.83); < 0.0001*	14.57 (14.06, 15.09); < 0.0001*	10.41 (9.96, 10.88); < 0.0001*	3.29 (3.18, 3.41); < 0.0001*
	Host absent net *vs* host present net	0.99 (0.94, 1.03); 1.0000	0.85 (0.81, 0.88); < 0.0001*	2.14 (2.06, 2.23); < 0.0001*	1.75 (1.60, 1.90); < 0.0001*
OS	Host absent net *vs* host absent flight/cone	1.93 (1.84, 2.02); < 0.0001*	0.76 (0.73, 0.79); < 0.0001*	5.61 (5.17, 6.08); < 0.0001*	29.21 (27.08, 31.50); < 0.0001*
	Host present net *vs* host present flight/cone	12.69 (12.11, 13.30); < 0.0001*	1.57 (1.48, 1.66); < 0.0001*	5.35 (5.12, 5.58); < 0.0001*	1.04 (1.00, 1.09); 0.0574
	Host absent net *vs* host present net	0.39 (0.37, 0.41); < 0.0001*	0.70 (0.66, 0.73); < 0.0001*	1.02 (0.96, 1.09); 1.0000	5.29 (4.98, 5.63); < 0.0001*

Multiple pairwise comparisons 95% Confidence Intervals and P-values corrected using the Bonferroni adjustment

*IS* insecticide susceptible, *IR* insecticide resistant, *UT* untreated net, *OS* olyset net, *P2* PermaNet 2.0 net

*Significant at 5% significance level

The presence of insecticide-treated material altered the observed behaviour of the two susceptible strains and, with the exception of Kisumu during exposure to P2, both strains had significantly higher net contact when the host was present (Table 1, Host absent net vs host present net P2: KS OR 0.99; 95% CI 0.94, 1.03; *P* = 1.0; NG OR 0.85; 95% CI 0.81, 0.88; *P* ≤ 0.0001; OS: KS OR 0.39; 95% CI 0.37, 0.41; *P* ≤ 0.0001; NG OR 0.70; 95% CI 0.66, 0.73; *P* ≤ 0.0001). This was not observed in resistant strains, which largely retained the host excitatory effects observed during untreated net tests (Table 1, Host absent net vs host present net P2: BF OR 2.14; 95% CI 2.06, 2.23; *P* ≤ 0.0001; VK7 OR 1.75; 95% CI 1.60, 1.90; *P* ≤ 0.0001; OS: BF OR 1.02; 95% CI 0.96, 1.09; *P* = 1.0; VK7 OR 5.29; 95% CI 4.98, 5.63; *P* ≤ 0.0001).

During P2 exposure there was a trend for *Anopheles* spp*.* mosquitoes to make less net contact compared to untreated net, indicative of a mild irritant effect. This effect was reduced in host tests (Table 2, Host absent P2 net vs UT net KS OR 1.00; 95% CI 0.96, 1.05; *P* = 1.0; NG OR 0.51; 95% CI 0.48, 0.53; *P* ≤ 0.0001; BF OR 0.92; 95% CI 0.88, 0.96; *P* ≤ 0.0001; VK7 OR 0.34; 95% CI 0.31, 0.38; *P* ≤ 0.0001 and Host present P2 net vs UT net KS OR 1.07; 95% CI 1.01, 1.13; *P* = 0.0061; NG OR 0.91; 95% CI 0.84, 0.99; *P* = 0.022; BF OR 1.29; 95% CI 1.23, 1.36; *P* ≤ 0.0001; VK7 OR 0.67; 95% CI 0.65, 0.70; *P* ≤ 0.0001; full multivariable analysis in Additional file 1: Table S5). In OS tests, mosquitoes of all strains made significantly less net contact compared to untreated net, regardless of the host presence (Table 2, Host absent OS net vs UT net KS OR 0.33; 95% CI 0.31, 0.35; *P* ≤ 0.0001; NG OR 0.14; 95% CI 0.13, 0.14; *P* ≤ 0.0001; BF OR 0.32; 95% CI 0.29, 0.34; *P* ≤ 0.0001; VK7 OR 0.58; 95% CI 0.55, 0.62; *P* ≤ 0.0001; Host present OS net vs UT net KS OR 0.88; 95% CI 0.84, 0.92; *P* ≤ 0.0001; NG OR 0.30; 95% CI 0.27, 0.33; *P* ≤ 0.0001; BF OR 0.92; 95% CI 0.88, 0.97; *P* ≤ 0.0001; VK7 OR 0.38; 95% CI 0.36, 0.40; *P* ≤ 0.0001).

**Table 2:** Treatment-Location comparisons within *An. gambiae s.l.* strain and host (present or absent) from a Beta-binomial Distribution model

Host	Comparison	Odds ratio (95% bonferroni adjusted confidence interval); bonferroni adjusted P-value			
		Kisumu (IS)	N’gousso (IS)	Banfora (IR)	VK7 (IR)
Absent	OS flight/cone *vs* P2 flight/cone	3.07 (2.94, 3.21); < 0.0001*	3.71 (3.55, 3.87); < 0.0001*	2.92 (2.73, 3.13); < 0.0001*	0.59 (0.53, 0.65); < 0.0001*
	OS flight/cone *vs* UT flight/cone	3.06 (2.90, 3.23); < 0.0001*	7.33 (7.03, 7.65); < 0.0001*	3.17 (2.95, 3.41); < 0.0001*	1.72 (1.61, 1.83); < 0.0001*
	OS net *vs* P2 Net	0.33 (0.31, 0.34); < 0.0001*	0.27 (0.26, 0.28); < 0.0001*	0.34 (0.32, 0.37); < 0.0001*	1.71 (1.53, 1.90); < 0.0001*
	OS net *vs* UT Net	0.33 (0.31, 0.35); < 0.0001*	0.14 (0.13, 0.14); < 0.0001*	0.32 (0.29, 0.34); < 0.0001*	0.58 (0.55, 0.62); < 0.0001*
	P2 net *vs* UT Net	1.00 (0.96, 1.05); 1.0000	0.51 (0.48, 0.53); < 0.0001*	0.92 (0.88, 0.96); < 0.0001*	0.34 (0.31, 0.38); < 0.0001*
Present	OS flight/cone *vs* P2 flight/cone	1.21 (1.14, 1.29); < 0.0001*	3.05 (2.89, 3.22); < 0.0001*	1.40 (1.33, 1.46); < 0.0001*	1.78 (1.70, 1.85); < 0.0001*
	OS flight/cone *vs* UT flight/Cone	1.14 (1.09, 1.19); < 0.0001*	3.34 (3.04, 3.66); < 0.0001*	1.08 (1.03, 1.14); < 0.0001*	2.64 (2.52, 2.76); < 0.0001*
	OS net *vs* P2 Net	0.82 (0.78, 0.87); < 0.0001*	0.33 (0.31, 0.35); < 0.0001*	0.72 (0.68, 0.75); < 0.0001*	0.56 (0.54, 0.59); < 0.0001*
	OS net *vs* UT Net	0.88 (0.84, 0.92); < 0.0001*	0.30 (0.27, 0.33); < 0.0001*	0.92 (0.88, 0.97); < 0.0001*	0.38 (0.36, 0.40); < 0.0001*
	P2 net *vs* UT Net	1.07 (1.01, 1.13); 0.0061*	0.91 (0.84, 0.99); 0.0222*	1.29 (1.23, 1.36); < 0.0001*	0.67 (0.65, 0.70); < 0.0001*

Multiple pairwise comparisons 95% Confidence Intervals and P-values corrected using the Bonferroni adjustment

*IS* insecticide susceptible, *IR* insecticide resistant, *UT* untreated net, *OS* olyset net, *P2* PermaNet 2.0 net

*Significant at 5% significance level

Comparing net contact at OS with P2 revealed differences in responses between the two pyrethroid-treated nets: significantly less net contact was observed during exposure to OS than P2 regardless of the host presence with the exception of VK7 in host absent tests (Table 2, Host absent OS net vs P2 net KS OR 0.33; 95% CI 0.31, 0.34; *P* ≤ 0.0001; NG OR 0.27; 95% CI 0.26, 0.28; *P* ≤ 0.0001; BF OR 0.34; 95% CI 0.32, 0.37; *P* ≤ 0.0001; VK7 OR 1.71; 95% CI 1.53, 1.90; *P* ≤ 0.0001; Host present OS net vs P2 net KS OR 0.82; 95% CI 0.78, 0.87; *P* ≤ 0.0001; NG OR 0.33; 95% CI 0.31, 0.35; *P* ≤ 0.0001; BF OR 0.72; 95% CI 0.68, 0.75; *P* ≤ 0.0001; VK7 OR 0.56; 95% CI 0.54, 0.59; *P* ≤ 0.0001).

### Impacts of exposure on life history traits

#### KD and 24 h mortality

VCT mortality was low (< 5%) in untreated net tests with the exception of the VK7 in host absent tests, where it reached 9%. Knockdown and 24 h mortality exceeded 90% in both susceptible strains following exposure to P2 and OS (Additional file 1: Table S6); mortality in resistant strains was 19% and 3% for Banfora and VK7, respectively, after P2 tests and 5% for both strains after OS tests (Additional file 1: Table S6).

#### Blood feeding and blood meal size

Due to mortality in the susceptible strains, analyses of recovery of blood feeding ability and blood meal size were only performed on resistant strains. After baseline untreated net tests, most Banfora and VK7 mosquitoes fed at 1-h post-exposure (Banfora: 85.7% (84/98), 93.8% (106/113); VK7: 86.7% (104/120), 93.5% (115/123) host absent and host present respectively, Additional file 1: Table S7); at 24 h, 78.6% (11/14) and 71% (5/7) of Banfora mosquitoes that did not feed at 1 h fed successfully in host absent and host present tests, respectively, and 12.5% (2/16) and 87.5% (7/8) of VK7 mosquitoes fed in host absent and host present tests (Additional file 1: Table S7).

Results from treated nets revealed the effects of treatment and host presence on blood feeding recovery. One hour after exposure, Banfora mosquitoes were significantly more likely to feed if the exposure was to untreated net compared to P2 and OS (Additional file 1: Table S7, Banfora 1 h Host absent OS vs UT OR 0.0349; 95% CI 0.0121, 0.1001, *P* ≤ 0.0001; Host present P2 vs UT OR 0.0077, 95% CI 0.0023, 0.0258, P ≤ 0.0001; OS vs UT OR 0.0533, 95% CI 0.0207, 0.1374; P ≤ 0.0001); after exposure to OS the presence of the host significantly increased chances of feeding at 1 h (Additional file 1: Table S8, Banfora 1 h OS Present vs Absent OR 7.1950, 95% CI 2.8809, 17.9689, P ≤ 0.0001). By 24 h the presence of the host during exposure no longer had an effect on blood feeding (Additional file 1: Table S8, Banfora 24 h P2 Present vs Absent OR 0.7062, 95% CI 0.2436, 2.0469, P = 0.5217; OS Present vs Absent OR 1.8564, 95% CI 0.6230, 5.5318, P = 0.2668).

At one hour post-exposure VK7 mosquitoes were also significantly more likely to feed after exposure to untreated net compared to P2, regardless of the presence of the host (Additional file 1: Table S7, VK7 1 h Host absent P2 vs UT OR 0.0177; 95% CI 0.0068, 0.0460, *P* ≤ 0.0001; Host present P2 vs UT OR 0.0110, 95% CI 0.0032, 0.0384, P ≤ 0.0001). However, a significant difference between feeding after untreated and OS exposure only occurred after host present tests (Additional file 1: Table S7, VK7 1 h Host absent OS vs UT OR 0.5464; 95% CI 0.2782, 1.0734, *P* = 0.0794; Host present OS vs UT OR 0.1115, 95% CI 0.0317, 0.3930, P = 0.0006). Unlike Banfora mosquitoes, the presence of the host did not have a significant effect on willingness to blood feed (Additional file 1: Table S8).

The mean blood meal size after untreated net tests was 12.67 µg/ml (SD = 6.65) and 13.25 µg/ml (SD = 6.39) for Banfora and VK7, respectively. No consistent effect of the host was observed in Banfora mosquitoes but in VK7 the host presence had a significant effect on the blood meal size, resulting in larger blood meals regardless of whether treated or untreated net was used (Additional file 1: Table S9, Host present vs Host Absent VK7 untreated mean haematin difference 2.86; 95% CI 1.25, 4.47; *P* = 0.0005; P2 mean haematin difference 2.26; 95% CI 0.06, 4.46; *P* = 0.0437; OS mean haematin difference 4.23; 95% CI 2.22, 6.24; *P* ≤ 0.0001). Blood meal sizes were consistently smaller after treated net exposures, although significantly so only in host absent tests for OS (Additional file 1: Table S10, Host absent OS vs Untreated Banfora mean difference − 5.29, 95% CI − 8.40, − 2.18; *P* = 0.0009; VK7 mean difference − 2.05, 95% CI − 3.48, − 0.62; *P* = 0.0049).

#### Longevity

The median longevity after VCT tests with untreated net was a minimum of 14 days for each strain, except for VK7 in host absent tests (Additional file 1: Table S11); accordingly, results for VK7 were only evaluated within each net treatment.

The median longevity of Banfora mosquitoes exposed to P2 was one and five days for host absent and host present tests, respectively (Additional file 1: Table S11, host absent Range 1–33; host present range 1–37). P2 had a relatively weak effect on the survival of Banfora mosquitoes: compared to those exposed to untreated nets, mosquitoes exposed to P2 were twice as likely to be dead by nine days (Additional file 1: Table S12, Banfora host absent P2 Hazard Ratio (HR) 2.03, 95% CI 1.40, 2.93, *P* = 0.0002; host present P2 HR 2.01, 95% CI 1.00, 4.06, *P* = 0.0510). Host effects on longevity were observed: Banfora and VK7 mosquitoes exposed to P2 with a host lived longer compared to tests without a host (Additional file 1: Table S13, P2 Banfora survival HR 0.64, 95% CI 0.49, 0.83, *P* = 0.0010; P2 VK7 host present HR 0.65, 95% CI 0.40, 1.06, *P* = 0.0836). Mosquitoes that blood fed and survived long enough to digest the blood meal did not exhibit significantly reduced longevity regardless of the presence of the host attractant (Additional file 1: Table S13, haematin Banfora HR 1.12, 95% CI 0.32, 3.87, *P* = 0.8587; VK7 HR 0.34, 95% CI 0.14, 0.86, *P* = 0.0231).

On average, Banfora mosquitoes exposed to OS died one day after exposure (Median = 1 day, Range 1–32 days). Compared to untreated net, Banfora mosquitoes exposed to OS were significantly more likely to die within 9 days, unless mosquitoes survived to take and digest a blood meal (Additional file 1: Table S12, Banfora host absent OS HR 1.66, 95% CI 1.09, 2.52, *P* = 0.0189; haematin HR 0.71, 95% CI 0.01, 39.39, *P* = 0.8667; host present OS HR 2.83, 95% CI 1.36, 5.87, *P* = 0.0053; haematin HR 1.45, 95% CI 0.53, 3.99, *P* = 0.4745).

VK7 mosquitoes exhibited reduced longevity after tests with a host compared to tests without a host, corresponding with the observed magnitude of net contact (Additional file 1: Table S13, VK7 OS host present HR 0.12; 95% CI 0.07, 0.20; *P* ≤ 0.0001; survival HR 0.09; 95% CI 0.04, 0.22; *P* ≤ 0.0001; Additional file 1: Table S4, VK7 host absent OS net vs OS flight OR 29.21; 95% CI 26.85, 31.77; *P* ≤ 0.0001; host present OS net vs OS flight OR 1.05; 95% CI 0.99, 1.09; *P* = 0.1436).

## Discussion

In the current malaria control landscape, where combating increases in insecticide resistance is an urgent priority, understanding the impacts of ITNs on local vector populations, especially in regions with different endemicities and varying uptakes of vector control tools, is of paramount importance [20, 34]. Previously reported characterizations of *An. gambiae s.l.* strains using free-flying mosquitoes revealed significant differences in mosquito behavioural responses at unbaited, human-baited untreated and ITNs [35]. These dissimilar responses, in addition to earlier studies showing that laboratory and field data do not always align [35, 36], suggest that evaluations of ITN efficacy would more accurately predict ‘real world’ performance if measures of vector behaviour were incorporated [12, 34].

The cone bioassay is one of WHO’s standard procedures for measuring the efficacy of ITNs and is widely implemented as part of routine malaria control practices across sub-Saharan Africa. However, the two endpoints, KD and 24-h mortality, are not designed to capture the effects of active ingredients beyond the rapid toxicity characteristic of pyrethroid exposure and are therefore unsuitable for characterizing the effects of nets co-formulated with active ingredients other than pyrethroids. The insights to be gained from extracting mosquito behaviour data with and without a host attractant during the cone test, with extended monitoring to capture life history traits were investigated.

Distinct differences were observed in the responses of *An. gambiae* to the host attractant and to ITN exposure. The host attractant elicited an excitatory effect, expressed as decreased net contact in the presence of the host, in all strains which was more pronounced in the two insecticide resistant strains compared to the two susceptible strains. This stimulative effect was preserved during the exposure of resistant strains to ITNs but lessened in the susceptible strains, suggesting a complex interplay between resistance status, host-seeking behaviours and ITN efficacy. This was further developed in our comparisons between two ITNs in widespread use treated with the same class of insecticide, Permanet 2.0 and Olyset. The behavioural profiles generated from the VCT were sensitive enough to clearly differentiate between the two ITNs and revealed that Permanet 2.0 elicited responses largely unaltered from those observed during testing with untreated net, whilst more pronounced irritative effects, i.e., significantly more time spent in flight, were observed with Olyset. Comparable profiles of both of these ITNs have been previously described using arm-in-cage (or similar) assays [39, 40], but until now it has not been possible to capture these data contemporaneously during routine efficacy testing.

ITN exposure reduced the likelihood of post-exposure blood feeding in both insecticide resistant strains, with an observed decrease in host absent tests from 86% blood feeding success after untreated netting tests to 0% and 11% in the Banfora strain after Permanet 2.0 and Olyset exposure, respectively, and in the VK7 strain from 87 to 12% after Permenet 2.0 exposure. In some cases, this reduction was ameliorated by exposure in the presence of a host, which significantly increased the chances of a mosquito taking a blood meal at one-hour post-exposure. This effect had disappeared by 24 h post-exposure; nevertheless, the magnitude of this effect suggests that post-exposure feeding should be incorporated into efficacy studies, especially for ITNs treated with AIs that might be expected to have a delayed effect on mortality.

The observed longevity following ITN exposure was also affected by the presence of a host during exposure: in the absence of blood feeding mosquitoes exposed to P2 with a host lived longer than those exposed without the host present. For those highly resistant mosquitoes that survived insecticide exposure long enough to feed and digest a blood meal, no delayed effects of longevity were observed, in keeping with previous studies and further suggesting the importance of measurements of blood feeding success when ITN efficacy is evaluated [11].

Although several behavioural trends that could be broadly classified into ‘susceptible’ or ‘resistant’ patterns were observed, strain-specific differences were apparent in some responses. Notably, the responses of the VK7 strain differed during exposure to OS compared to the other strains. Both resistant strains tested in this study originate from Southwest Burkina Faso and were colonized during the same year. The high pyrethroid resistance phenotype in each strain is partially conferred by both target site and metabolic resistance but each strain has additional resistance mechanisms; notably, resistance in the Banfora strain is partially conferred by elevated levels of sensory appendage protein, SAP2 and is also associated with elevated rates of respiration whereas elevated levels of gene families putatively involved in pyrethroid sequestration are found in the VK7 strain [23, 25, 37, 38]. It would be instructive to repeat this study using *Anopheles* strains carrying moderate pyrethroid resistance and/or single mechanisms of resistance.

As has been previously noted, the smaller scale of test arenas and standardized environmental conditions in most laboratories contributes to discrepancies found between laboratory and field studies [35, 41–47]. Whilst the shape and volume of the WHO cone undoubtedly create artificial conditions of near-forced net contact, it is encouraging to note that the excitatory effect provoked by the addition of a host captures at a small scale the finding from Parker et al. that *An. gambiae* activity at an untreated net was significantly lower in the absence of human bait. This indicates that data more representative of those typically obtained from field-scale experiments can be collected in the laboratory using appropriately modified assays.

Using scan sampling to translate mosquito activity in the cone into quantified behavioural composites has proved very informative but has its limitations. For example, the static nature of scan sampling does not allow determination of whether time spent in contact with the net is spent resting or in active host seeking, information that would be useful in understanding the apparent lessening of the host effect on susceptible mosquitoes. Furthermore, although the addition of the video recording does not appreciably increase the time required to perform laboratory experiments, subsequent scan sampling adds additional analysis time. It is possible that this could be mitigated by applying an automated video analysis method to record mosquito positions.

## Conclusions

The results demonstrate that the WHO cone test, with appropriate adaptations and life history trait monitoring, can be used to construct behavioural composites for standard ITNs that are more informative for routine efficacy testing than the standard cone test method. Adding a host to the test reproduces at a small scale behavioural modes that can usually only be observed in larger scale tests; even in the three-minute test window these alterations in mosquito-host-ITN interactions yield knock-on effects to post-exposure blood feeding and subsequent survival that are directly applicable to efficacy evaluations in local vector populations. Such data could prove invaluable in evaluating the success of combination nets currently being rolled out across Africa.

## Supplementary Information

**Additional file 1**: **Table S1.** Total imprecision assessment of VCT on untreated net by event type (net contact, flight, cone contact) for each mosquito strain (Kisumu, N’gousso, Banfora and VK7) of *An. gambiae s.l.* in the absence and presence of a host. Where n (mos) = number of mosquitoes per group, n (reps) = number of replicates performed per group, mean = mean number of mosquitoes per event, SD = standard deviation of the mean, LCLM = lower confidence level mean, UCLM = upper confidence level mean, %CV = percentage coefficient of variation, IS = insecticide susceptible, and IR = insecticide resistant. **Table S2.** Within-day imprecision assessment of VCT on untreated net by event type (net contact, flight, cone contact) for each strain (Kisumu, N’gousso, Banfora and VK7) of *An. gambiae s.l.* in the absence and presence of a host. Where n (test days) = number of test days per group, n (reps per test day) = number of replicates performed per test day per group, mean = mean number of mosquitoes per event, SD = standard deviation of the mean, %CV = percentage coefficient of variation, IS = insecticide susceptible, and IR = insecticide resistant. **Table S3.** Variation in test stages. Assessment of VCT on untreated net by event type (net contact only) for each strain (Kisumu, N’gousso, Banfora and VK7) of *An. gambiae s.l.* in the absence and presence of a host. Where n (reps) = number of replicates performed per group, %CV = percentage coefficient of variation by time (s = seconds), IS = insecticide susceptible, and IR = insecticide resistant. **Table S4.** Host-Location comparisons within *An. gambiae s.l.* strain and net treatment. A Beta-binomial Distribution model fitted**.** Multiple pairwise comparisons 95% Confidence Intervals and P-values corrected using the Bonferroni adjustment. Where IS = insecticide susceptible, IR = insecticide resistant, UT = Untreated net, OS = Olyset net, P2 = PermaNet 2.0 net and * = Significant at 5% significance level. **Table S5.** Treatment-Location comparisons within *An. gambiae* strain (Kisumu, N’gousso, Banfora and VK7) and host (present or absent). A Beta-binomial Distribution model Results**.** Multiple pairwise comparisons 95% Confidence Intervals and P-values corrected using the Bonferroni adjustment. Where IS = insecticide susceptible, IR = insecticide resistant, UT = Untreated net, OS = Olyset net, P2 = PermaNet 2.0 net and * = Significant at 5% significance level. **Table S6.** Knock-down (KD) (1 h) and mortality (24 h) after net exposure for insecticide susceptible (Kisumu and N’gousso) and insecticide resistant (VK7 and Banfora) strains of *An. gambiae s.l.* against PermaNet 2.0 (P2) and Olyset (OS) ITNs, in VCT validation experiments. **Table S7.** Treatment comparisons—Willingness to refeed at 1 or 24 h within *An. gambiae* strain and host (present or absent). A Binary Logistic Regression model fitted using Generalised Estimating Equation. Where IR = insecticide resistant, UT = Untreated net, OS = Olyset net, P2 = PermaNet 2.0 net and * = Significant at 5% significance level. **Table S8.** Host comparisons—Willingness to refeed at 1 or 24 h within *An. gambiae* strain and treatment. A Binary Logistic Regression model fitted using Generalised Estimating Equation. Where IR = insecticide resistant, UT = Untreated net, OS = Olyset net, P2 = PermaNet 2.0 net and * = Significant at 5% significance level. **Table S9.** Treatment comparisons – Blood meal size within *An. gambiae* strain and host (present or absent). A Linear Regression model fitted using Generalised Estimating Equation. Where IS = insecticide susceptible, IR = insecticide resistant, UT = Untreated net, OS = Olyset net, P2 = PermaNet 2.0, CI = Confidence Interval net and * = Significant at 5% significance level. **Table S10.** Host comparisons – Blood meal size within *An. gambiae* strain and treatment. A Linear Regression model fitted using Generalised Estimating Equation. Where IS = insecticide susceptible, IR = insecticide resistant, UT = Untreated net, OS = Olyset net, P2 = PermaNet 2.0 net and * = Significant at 5% significance level. **Table S11.** Descriptive statistics for longevity data within host, *An. gambiae* strain and net treatment. Where BF = Banfora, VK = VK7, UT = Untreated net, P2 = PermaNet 2.0 net, OS = Olyset net, IR = insecticide resistant, n = sample size, % = percentage, SD = Standard Deviation, Min = Minimum, Max = Maximum, CI = Confidence Interval, Yes n = number of mosquitoes that died within 9 days and * results for mosquitoes that died within 9 days post-exposure only. **Table S12.** Net treatment comparisons—Mortality within 9 days within *An. gambiae* strain and host. A Weighted Cox Regression Model fitted adjusted for other predictors (time fed, wingspan, haematin and net proportion). Where UT = Untreated net, P2 = PermaNet 2.0 net, OS = Olyset net, IR = insecticide resistant, Ref = Reference group, Net proportion = Mean proportion of mosquitoes on the net during exposure (behaviour data) and * = Significant at 5% significance level. Censoring at 9 days. **Table S13.** Host comparisons—Mortality within 9 days within *An. gambiae* strain and net treatment. A Weighted Cox Regression Model fitted adjusted for other predictors (time fed, wingspan, haematin and net proportion). Where UT = Untreated net, P2 = PermaNet 2.0 net, OS = Olyset net, IR = insecticide resistant, Ref = Reference group, Net proportion = Mean proportion of mosquitoes on the net during exposure (behaviour data) and * = Significant at 5% significance level. Censoring at 9 days.

## Data Availability

The data generated and/or analysed during the current study are available from the corresponding author on reasonable request.
