# Supplementary material for: A closer look at the WHO cone bioassay: video analysis of the hidden effects of a human host on mosquito behaviour and insecticide contact
Source: Malar J. 2022 Jul 1;21:208. doi: 10.1186/s12936-022-04232-4 (PMC9248144; doi:10.1186/s12936-022-04232-4)
Supplement: Supplementary file 1 — Additional file 1: Table S1. Total imprecision assessment of VCT on untreated net by event type (net contact, flight, cone contact) for each mosquito strain (Kisumu, N’gousso, Banfora and VK7) of An. gambiae s.l. in the absence and presence of a host. Where n (mos) = number of mosquitoes per group, n (reps) = number of replicates performed per group, mean = mean number of mosquitoes per event, SD = standard deviation of the mean, LCLM = lower confidence level mean, UCLM = upper confidence level mean, %CV = percentage coefficient of variation, IS = insecticide susceptible, and IR = insecticide resistant. Table S2. Within-day imprecision assessment of VCT on untreated net by event type (net contact, flight, cone contact) for each strain (Kisumu, N’gousso, Banfora and VK7) of An. gambiae s.l. in the absence and presence of a host. Where n (test days) = number of test days per group, n (reps per test day) = number of replicates performed per test day per group, mean = mean number of mosquitoes per event, SD = standard deviation of the mean, %CV = percentage coefficient of variation, IS = insecticide susceptible, and IR = insecticide resistant. Table S3. Variation in test stages. Assessment of VCT on untreated net by event type (net contact only) for each strain (Kisumu, N’gousso, Banfora and VK7) of An. gambiae s.l. in the absence and presence of a host. Where n (reps) = number of replicates performed per group, %CV = percentage coefficient of variation by time (s = seconds), IS = insecticide susceptible, and IR = insecticide resistant. Table S4. Host-Location comparisons within An. gambiae s.l. strain and net treatment. A Beta-binomial Distribution model fitted. Multiple pairwise comparisons 95% Confidence Intervals and P-values corrected using the Bonferroni adjustment. Where IS = insecticide susceptible, IR = insecticide resistant, UT = Untreated net, OS = Olyset net, P2 = PermaNet 2.0 net and * = Significant at 5% significance level. Table S5. Treatment-Location com [file 12936_2022_4232_MOESM1_ESM.docx]

**Additional file 1**

**Supplementary Tables and Legends**

**Summary**

Table S1. Total imprecision assessment

Table S2. Within-day imprecision assessment

Table S3. Variation in test stage

Table S4. Host-Location comparisons within *An. gambiae* *s.l.* strain and net treatment.

Table S5. Treatment-Location comparisons within *An. gambiae* strain (Kisumu, N’gousso, Banfora and VK7) and host (present or absent)

Table S6. Knock-down (KD) (1h) and mortality (24h)

Table S7. Treatment comparisons - Willingness to refeed at 1 or 24 hours within *An. gambiae* strain and host (present or absent)

Table S8. Host comparisons - Willingness to refeed at 1 or 24 hours within *An. gambiae* strain and treatment

Table S9. Treatment comparisons – Blood meal size within *An. gambiae* strain and host (present or absent).

Table S10. Host comparisons – Blood meal size within *An. gambiae* strain and treatment

Table S11. Descriptive statistics for longevity data within host, *An. gambiae* strain and net treatment

Table S12. Net treatment comparisons - Mortality within 9 days within *An. gambiae* strain and host

Table S13. Host comparisons - Mortality within 9 days within *An. gambiae* strain and net treatment

| **Event** | **Host absent** | | | | |  | **Host present** | | | | |
| --- | --- | --- | --- | --- | --- | --- | --- | --- | --- | --- | --- |
|  | **n (mos)**  **n (reps)** | **Mean** | **SD** | **LCLM-UCLM** | **%CV** |  | **n (mos)**  **n (reps)** | **Mean** | **SD** | **LCLM-UCLM** | **%CV** |
| **Net** |  |  |  |  |  |  |  |  |  |  |  |
| Kisumu  (IS) | 125  (25) | 4.05 | 0.98 | 3.99-4.12 | 24.19 |  | 130  (26) | 4.08 | 0.90 | 4.02-4.14 | 22.04 |
| N’gousso (IS) | 115  (23) | 4.43 | 0.76 | 4.38-4.48 | 17.11 |  | 100  (20) | 4.11 | 1.23 | 4.02-4.20 | 30.04 |
| Banfora (IR) | 105  (21) | 4.52 | 0.69 | 4.50-4.57 | 15.12 |  | 115  (23) | 3.55 | 1.06 | 3.48-3.62 | 29.90 |
| VK7  (IR) | 120  (24) | 4.65 | 0.63 | 4.61-4.69 | 13.56 |  | 125  (25) | 3.74 | 0.98 | 3.67-3.80 | 26.19 |
| **Flight** |  |  |  |  |  |  |  |  |  |  |  |
| Kisumu  (IS) | 125  (25) | 0.88 | 0.97 | 0.82-0.94 | 110.10 |  | 130  (26) | 0.81 | 0.87 | 0.75-0.86 | 108.09 |
| N’gousso (IS) | 115  (23) | 0.48 | 0.74 | 0.43-0.53 | 153.72 |  | 100  (20) | 0.61 | 0.79 | 0.56-0.67 | 129.12 |
| Banfora (IR) | 105  (21) | 0.43 | 0.66 | 0.39-0.48 | 152.78 |  | 115  (23) | 1.35 | 1.04 | 1.28-1.42 | 77.37 |
| VK7  (IR) | 120  (24) | 0.25 | 0.57 | 0.22-0.29 | 223.32 |  | 125  (25) | 0.96 | 0.93 | 0.90-1.02 | 97.27 |
| **Cone** |  |  |  |  |  |  |  |  |  |  |  |
| Kisumu  (IS) | 125  (25) | 0.06 | 0.27 | 0.05-0.08 | 422.92 |  | 130  (26) | 0.11 | 0.35 | 0.09-0.14 | 304.84 |
| N’gousso (IS) | 115  (23) | 0.08 | 0.28 | 0.06-0.10 | 336.70 |  | 100  (20) | 0.28 | 0.97 | 0.21-0.35 | 349.36 |
| Banfora (IR) | 105  (21) | 0.04 | 0.21 | 0.03-0.06 | 467.77 |  | 115  (23) | 0.10 | 0.32 | 0.07-0.12 | 336.68 |
| VK7  (IR) | 120  (24) | 0.10 | 0.34 | 0.08-0.12 | 346.93 |  | 125  (25) | 0.30 | 0.54 | 0.27-0.34 | 178.48 |

**Table S1. Total imprecision assessment** of VCT on untreated net by event type (net contact, flight, cone contact) for each mosquito strain (Kisumu, N’gousso, Banfora and VK7) of *An. gambiae s.l.* in the absence and presence of a host. Where n (mos) = number of mosquitoes per group, n (reps) = number of replicates performed per group, mean = mean number of mosquitoes per event, SD = standard deviation of the mean, LCLM = lower confidence level mean, UCLM = upper confidence level mean, %CV = percentage coefficient of variation, IS = insecticide susceptible, and IR = insecticide resistant.

| **Event** | **Host absent** | | | | |  | **Host present** | | | | |
| --- | --- | --- | --- | --- | --- | --- | --- | --- | --- | --- | --- |
|  | **n**  **(test days)** | **Range**  **(reps per test day)** | **Range (Mean)** | **Range**  **(SD)** | **Range (%CV)** |  | **n**  **(test days)** | **Range**  **(reps per test day)** | **Range (Mean)** | **Range**  **(SD)** | **Range (%CV)** |
| **Net** |  |  |  |  |  |  |  |  |  |  |  |
| Kisumu  (IS) | 4 | 2-14 | 3.27-4.38 | 0.84-0.96 | 19.29-29.43 |  | 7 | 1-6 | 3.7-4.73 | 0.53-0.96 | 11.23-26.14 |
| N’gousso  (IS) | 5 | 2-8 | 4.03-4.76 | 0.54-0.95 | 11.42-23.55 |  | 5 | 1-9 | 3.87-4.81 | 0.39-1.54 | 8.25-39.64 |
| Banfora  (IR) | 3 | 4-10 | 4.13-4.68 | 0.57-0.76 | 12.07-18.37 |  | 4 | 3-8 | 3.22-4.12 | 0.89-1.13 | 21.88-32.99 |
| VK7  (IR) | 3 | 4-10 | 4.61-4.75 | 0.55-0.71 | 11.85-15.35 |  | 5 | 1-10 | 3.45-4.30 | 0.85-1.00 | 19.67-27.08 |
| **Flight** |  |  |  |  |  |  |  |  |  |  |  |
| Kisumu  (IS) | 4 | 2-14 | 0.60-1.73 | 0.81-0.97 | 55.65-139.52 |  | 7 | 1-6 | 0.20-1.30 | 0.47-0.96 | 74.61-230.67 |
| N’gousso  (IS) | 5 | 2-8 | 0.24-0.97 | 0.54-0.95 | 97.46-223.23 |  | 5 | 1-9 | 0.19-1.03 | 0.40-0.93 | 77.78-209.88 |
| Banfora  (IR) | 3 | 4-10 | 0.30-0.86 | 0.55-0.76 | 89.15-186.38 |  | 4 | 3-8 | 0.85-1.67 | 0.88-1.12 | 72.74-103.43 |
| VK7  (IR) | 3 | 4-10 | 0.14-0.39 | 0.41-0.70 | 179.44-289.39 |  | 5 | 1-10 | 0.35-1.29 | 0.68-1.00 | 69.52-194.76 |
| **Cone** |  |  |  |  |  |  |  |  |  |  |  |
| Kisumu (IS) | 4 | 2-14 | 0-0.45 | 0-0.55 | 0-697.58 |  | 7 | 1-6 | 0-0.19 | 0-0.63 | 129.60-555.45 |
| N’gousso (IS) | 5 | 2-8 | 0-0.22 | 0-0.42 | 0-860.23 |  | 5 | 1-9 | 0-0.52 | 0-0.45 | 0-674.51 |
| Banfora (IR) | 3 | 4-10 | 0.01-0.10 | 0.12-0.30 | 306.5-857.30 |  | 4 | 3-8 | 0-0.16 | 0-0.41 | 0-547.68 |
| VK7  (IR) | 3 | 4-10 | 0-0.21 | 0.07-0.44 | 203.37-1358.30 |  | 5 | 1-10 | 0-0.59 | 0-0.75 | 0-251.78 |

**Table S2. Within-day imprecision assessment** of VCT on untreated net by event type (net contact, flight, cone contact) for each strain (Kisumu, N’gousso, Banfora and VK7) of *An. gambiae s.l.* in the absence and presence of a host. Where n (test days) = number of test days per group, n (reps per test day) = number of replicates performed per test day per group, mean = mean number of mosquitoes per event, SD = standard deviation of the mean, %CV = percentage coefficient of variation, IS = insecticide susceptible, and IR = insecticide resistant.

| **Event** | **Host absent** | | | | | | |  | **Host present** | | | | | | |
| --- | --- | --- | --- | --- | --- | --- | --- | --- | --- | --- | --- | --- | --- | --- | --- |
|  | **n (reps)** | **Range (%CV) by time** | | | | | |  | **n**  **(reps)** | **Range (%CV) by time** | | | | | |
|  |  | **0-30s** | **35-60s** | **65-90s** | **95-120s** | **125-150s** | **155-180s** |  |  | **0-30s** | **35-60s** | **65-90s** | **95-120s** | **125-150s** | **155-180s** |
| **Net** |  |  |  |  |  |  |  |  |  |  |  |  |  |  |  |
| Kisumu  (IS) | 25 | 21.23  30.39 | 20.74  37.49 | 19.23  28.30 | 22.00  26.77 | 18.18  25.24 | 16.05  26.47 |  | 26 | 17.29  23.96 | 19.12  26.20 | 15.62  25.26 | 16.55  21.57 | 19.27  25.71 | 19.53  28.77 |
| N’gousso  (IS) | 23 | 14.24  23.20 | 16.42  22.61 | 14.94  22.37 | 12.31  24.70 | 10.47  19.10 | 12.65  17.65 |  | 20 | 29.15  34.34 | 26.13  39.53 | 26.13  36.24 | 27.30  33.17 | 26.25  33.48 | 29.54  34.41 |
| Banfora  (IR) | 21 | 13.07  22.30 | 13.30  19.72 | 13.07  22.30 | 8.40  14.50 | 7.38  15.64 | 6.13  12.37 |  | 23 | 26.89  35.58 | 25.06  37.45 | 21.66  29.90 | 21.68  38.09 | 25.15  34.13 | 27.83  36.56 |
| VK7  (IR) | 24 | 11.68  30.74 | 9.31  16.92 | 8.66  15.65 | 5.74  10.69 | 7.88  12.10 | 7.88  24.73 |  | 25 | 19.54  26.32 | 25.29  36.08 | 23.39  32.08 | 19.23  30.57 | 22.44  31.32 | 21.49  27.46 |

**Table S3. Variation in test stages.** Assessment of VCT on untreated net by event type (net contact only) for each strain (Kisumu, N’gousso, Banfora and VK7) of *An. gambiae s.l.* in the absence and presence of a host. Where n (reps) = number of replicates performed per group, %CV = percentage coefficient of variation by time (s = seconds), IS = insecticide susceptible, and IR = insecticide resistant.

| **Net** | **Comparison** | **Odds Ratio (95% Bonferroni Adjusted Confidence Interval); Bonferroni Adjusted P-value** | | | |
| --- | --- | --- | --- | --- | --- |
|  |  | **Kisumu** | **N’gousso** | **Banfora** | **VK7** |
| **UT** | Host Absent Net *vs*  Host Absent Flight/Cone | 18.07  (17.16, 19.03); <.0001 * | 40.79  (39.18, 42.46); <.0001 * | 56.43  (54.12, 58.85); <.0001 * | 86.26  (83.11, 89.53); <.0001 * |
|  | Host Present Net *vs*  Host Present Flight/Cone | 16.41  (15.83, 17.01); <.0001 * | 17.44  (15.78, 19.27); <.0001 * | 6.25  (5.96, 6.56); <.0001 * | 7.25  (6.98, 7.53); <.0001 * |
|  | Host Absent Net *vs*  Host Present Net | 1.05  (1.00, 1.10); 0.0256 * | 1.53  (1.42, 1.65); <.0001 * | 3.01  (2.87, 3.14); <.0001 * | 3.45  (3.32, 3.58); <.0001 * |
|  | Host Absent Flight/Cone *vs*  Host Present Net | 0.058  (0.056, 0.061); <.0001 * | 0.038  (0.035, 0.040); <.0001 * | 0.053  (0.051, 0.056); <.0001 * | 0.040  (0.039, 0.042); <.0001 * |
|  | Host Absent Net *vs*  Host Present Flight/Cone | 17.22  (16.47, 18.01); <.0001 * | 26.67  (24.71, 28.78); <.0001 * | 18.78  (17.95, 19.64); <.0001 * | 25.01  (24.09, 25.96); <.0001 * |
|  | Host Absent Flight/Cone *vs*  Host Present Flight/Cone | 0.95  (0.91, 1.00); 0.0256 * | 0.65  (0.61, 0.71); <.0001 * | 0.33  (0.32, 0.35); <.0001 * | 0.29  (0.28, 0.30); <.0001 * |
| **P2** | Host Absent Net *vs*  Host Absent Flight/Cone | 18.22  (17.66, 18.81); <.0001 * | 10.44  (10.01, 10.89); <.0001 * | 47.85  (46.29, 49.46); <.0001 * | 10.02  (8.92, 11.26); <.0001 * |
|  | Host Present Net *vs*  Host Present Flight/Cone | 18.69  (17.63, 19.83); <.0001 * | 14.57  (14.06, 15.09); <.0001 * | 10.41  (9.96, 10.88); <.0001 * | 3.29  (3.18, 3.41); <.0001 * |
|  | Host Absent Net *vs*  Host Present Net | 0.99  (0.94, 1.03); 1.0000 | 0.85  (0.81, 0.88); <.0001 * | 2.14  (2.06, 2.23); <.0001 * | 1.75  (1.60, 1.90); <.0001 * |
|  | Host Absent Flight/Cone *vs*  Host Present Net | 0.054  (0.052, 0.057); <.0001 * | 0.081  (0.078, 0.084); <.0001 * | 0.045  (0.043, 0.047); <.0001 * | 0.174  (0.160, 0.190); <.0001 * |
|  | Host Absent Net *vs*  Host Present Flight/Cone | 18.46  (17.61, 19.35); <.0001 * | 12.33  (11.87, 12.82); <.0001 * | 22.32  (21.46, 23.21); <.0001 * | 5.74  (5.27, 6.26); <.0001 * |
| **P2 cont.** | Host Absent Flight/Cone *vs*  Host Present Flight/Cone | 1.01  (0.97, 1.06); 1.0000 | 1.18  (1.14, 1.23); <.0001 * | 0.47  (0.45, 0.48); <.0001 * | 0.57  (0.53, 0.62); <.0001 * |
| **OS** | Host Absent Net *vs*  Host Absent Flight/Cone | 1.93  (1.84, 2.02); <.0001 * | 0.76  (0.73, 0.79); <.0001 * | 5.61  (5.17, 6.08); <.0001 * | 29.21  (27.08, 31.50); <.0001 * |
|  | Host Present Net *vs*  Host Present Flight/Cone | 12.69  (12.11, 13.30); <.0001 * | 1.57  (1.48, 1.66); <.0001 * | 5.35  (5.12, 5.58); <.0001 * | 1.04  (1.00, 1.09); 0.0574 |
|  | Host Absent Net *vs*  Host Present Net | 0.39  (0.37, 0.41); <.0001 * | 0.70  (0.66, 0.73); <.0001 * | 1.02  (0.96, 1.09); 1.0000 | 5.29  (4.98, 5.63); <.0001 * |
|  | Host Absent Flight/Cone *vs*  Host Present Net | 0.20  (0.19, 0.21); <.0001 * | 0.92  (0.87, 0.96); <.0001 * | 0.18  (0.17, 0.19); <.0001 * | 0.18  (0.17, 0.19); <.0001 * |
|  | Host Absent Net *vs*  Host Present Flight/Cone | 4.95  (4.73, 5.19); <.0001 * | 1.09  (1.04, 1.14); <.0001 * | 5.47  (5.13, 5.84); <.0001 * | 5.52  (5.19, 5.86); <.0001 * |
|  | Host Absent Flight/Cone *vs*  Host Present Flight/Cone | 2.56  (2.45, 2.69); <.0001 * | 1.44  (1.37, 1.51); <.0001 * | 0.98  (0.91, 1.04); 1.0000 | 0.19  (0.18, 0.20); <.0001 * |

**Table S4. Host-Location comparisons within *An. gambiae* *s.l.* strain and net treatment.** A Beta-binomial Distribution model fitted**.** Multiple pairwise comparisons 95% Confidence Intervals and P-values corrected using the Bonferroni adjustment. Where IS = insecticide susceptible, IR = insecticide resistant, UT = Untreated net, OS = Olyset net, P2 = PermaNet 2.0 net and * = Significant at 5% significance level.

|  | | **Odds Ratio (95% Bonferroni Adjusted Confidence Interval); Bonferroni Adjusted P-value** | | | |
| --- | --- | --- | --- | --- | --- |
| **Host** | **Comparison** | **Kisumu** | **N’gousso** | **Banfora** | **VK7** |
| **Absent** | OS Flight/Cone *vs*  P2 Flight/Cone | 3.07  (2.94, 3.21); <.0001 * | 3.71  (3.55, 3.87); <.0001 * | 2.92  (2.73, 3.13); <.0001 * | 0.59  (0.53, 0.65); <.0001 * |
|  | OS Flight/Cone *vs*  UT Flight/Cone | 3.06  (2.90, 3.23); <.0001 * | 7.33  (7.03, 7.65); <.0001 * | 3.17  (2.95, 3.41); <.0001 * | 1.72  (1.61, 1.83); <.0001 * |
|  | OS Net *vs*  OS Flight/Cone | 1.93  (1.84, 2.03); <.0001 * | 0.76  (0.73, 0.79); <.0001 * | 5.61  (5.12, 6.14); <.0001 * | 29.21  (26.85, 31.77); <.0001 * |
|  | OS Net *vs*  P2 Flight/Cone | 5.93  (5.68, 6.20); <.0001 * | 2.81  (2.70, 2.94); <.0001 * | 16.38  (15.28, 17.56); <.0001 * | 17.11  (15.34, 19.09); <.0001 * |
|  | OS Net *vs*  P2 Net | 0.33  (0.31, 0.34); <.0001 * | 0.27  (0.26, 0.28); <.0001 * | 0.34  (0.32, 0.37); <.0001 * | 1.71  (1.53, 1.90); <.0001 * |
|  | OS Net *vs*  UT Flight/Cone | 5.91  (5.59, 6.24); <.0001 * | 5.56  (5.33, 5.80); <.0001 * | 17.79  (16.55, 19.12); <.0001 * | 50.19  (46.97, 53.63); <.0001 * |
|  | OS Net *vs*  UT Net | 0.33  (0.31, 0.35); <.0001 * | 0.14  (0.13, 0.14); <.0001 * | 0.32  (0.29, 0.34); <.0001 * | 0.58  (0.55, 0.62); <.0001 * |
|  | P2 Flight/Cone *vs*  UT Flight/Cone | 1.00  (0.95, 1.04); 1.0000 | 1.98  (1.89, 2.07); <.0001 * | 1.09  (1.04, 1.13); <.0001 * | 2.93  (2.67, 3.23); <.0001 * |
|  | P2 Net *vs*  OS Flight/Cone | 5.93  (5.68, 6.20); <.0001 * | 2.81  (2.70, 2.94); <.0001 * | 16.38  (15.28, 17.56); <.0001 * | 17.11  (15.34, 19.09); <.0001 * |
|  | P2 Net *vs*  P2 Flight/Cone | 18.22  (17.59, 18.88); <.0001 * | 10.44  (9.97, 10.94); <.0001 * | 47.85  (46.12, 49.65); <.0001 * | 10.02  (8.81, 11.41); <.0001 * |
|  | P2 Net *vs*  UT Flight/Cone | 18.15  (17.30, 19.04); <.0001 * | 20.64  (19.71, 21.60); <.0001 * | 51.97  (49.82, 54.20); <.0001 * | 29.41  (26.70, 32.38); <.0001 * |
| **Absent cont.** | P2 Net *vs*  UT Net | 1.00  (0.96, 1.05); 1.0000 | 0.51  (0.48, 0.53); <.0001 * | 0.92  (0.88, 0.96); <.0001 * | 0.34  (0.31, 0.38); <.0001 * |
|  | UT Net *vs*  OS Flight/Cone | 5.91  (5.59, 6.24); <.0001 * | 5.56  (5.33, 5.80); <.0001 * | 17.79  (16.55, 19.12); <.0001 * | 50.19  (46.97, 53.63); <.0001 * |
|  | UT Net *vs*  P2 Flight/Cone | 18.15  (17.30, 19.04); <.0001 * | 20.64  (19.71, 21.60); <.0001 * | 51.97  (49.82, 54.20); <.0001 * | 29.41  (26.70, 32.38); <.0001 * |
|  | UT Net *vs*  UT Flight/Cone | 18.07  (17.05, 19.15); <.0001 * | 40.79  (39.00, 42.65); <.0001 * | 56.43  (53.86, 59.13); <.0001 * | 86.26  (82.76, 89.90); <.0001 * |
| **Present** | OS Flight/Cone *vs*  P2 Flight/Cone | 1.21  (1.14, 1.29); <.0001 * | 3.05  (2.89, 3.22); <.0001 * | 1.40  (1.33, 1.46); <.0001 * | 1.78  (1.70, 1.85); <.0001 * |
|  | OS Flight/Cone *vs*  UT Flight/Cone | 1.14  (1.09, 1.19); <.0001 * | 3.34  (3.04, 3.66); <.0001 * | 1.08  (1.03, 1.14); <.0001 * | 2.64  (2.52, 2.76); <.0001 * |
|  | OS Net *vs*  OS Flight/Cone | 12.69  (12.05, 13.37); <.0001 * | 1.57  (1.47, 1.67); <.0001 * | 5.35  (5.10, 5.61); <.0001 * | 1.04  (0.99, 1.09); 0.1436 |
|  | OS Net *vs*  P2 Flight/Cone | 15.40  (14.52, 16.34); <.0001 * | 4.78  (4.52, 5.05); <.0001 * | 7.46  (7.11, 7.83); <.0001 * | 1.85  (1.77, 1.93); <.0001 * |
|  | OS Net *vs*  P2 Net | 0.82  (0.78, 0.87); <.0001 * | 0.33  (0.31, 0.35); <.0001 * | 0.72  (0.68, 0.75); <.0001 * | 0.56  (0.54, 0.59); <.0001 * |
|  | OS Net *vs*  UT Flight/Cone | 14.43  (13.78, 15.12); <.0001 * | 5.23  (4.77, 5.73); <.0001 * | 5.78  (5.49, 6.08); <.0001 * | 2.75  (2.63, 2.87); <.0001 * |
|  | OS Net *vs*  UT Net | 0.88  (0.84, 0.92); <.0001 * | 0.30  (0.27, 0.33); <.0001 * | 0.92  (0.88, 0.97); <.0001 * | 0.38  (0.36, 0.40); <.0001 * |
| **Present cont.** | P2 Flight/Cone *vs*  UT Flight/Cone | 0.94  (0.89, 0.99); 0.0061 * | 1.09  (1.01, 1.19); 0.0222 * | 0.77  (0.74, 0.82); <.0001 * | 1.48  (1.43, 1.55); <.0001 * |
|  | P2 Net *vs*  OS Flight/Cone | 15.40  (14.52, 16.34); <.0001 * | 4.78  (4.52, 5.05); <.0001 * | 7.46  (7.11, 7.83); <.0001 * | 1.85  (1.77, 1.93); <.0001 * |
|  | P2 Net *vs*  P2 Flight/Cone | 18.69  (17.51, 19.96); <.0001 * | 14.57  (14.01, 15.15); <.0001 * | 10.41  (9.91, 10.94); <.0001 * | 3.29  (3.16, 3.42); <.0001 * |
|  | P2 Net *vs*  UT Flight/Cone | 17.52  (16.59, 18.49); <.0001 * | 15.94  (14.66, 17.33); <.0001 * | 8.07  (7.66, 8.49); <.0001 * | 4.88  (4.69, 5.08); <.0001 * |
|  | P2 Net *vs*  UT Net | 1.07  (1.01, 1.13); 0.0061 * | 0.91  (0.84, 0.99); 0.0222 * | 1.29  (1.23, 1.36); <.0001 * | 0.67  (0.65, 0.70); <.0001 * |
|  | UT Net *vs*  OS Flight/Cone | 14.43  (13.78, 15.12); <.0001 * | 5.23  (4.77, 5.73); <.0001 * | 5.78  (5.49, 6.08); <.0001 * | 2.75  (2.63, 2.87); <.0001 * |
|  | UT Net *vs*  P2 Flight/Cone | 17.52  (16.59, 18.49); <.0001 * | 15.94  (14.66, 17.33); <.0001 * | 8.07  (7.66, 8.49); <.0001 * | 4.88  (4.69, 5.08); <.0001 * |
|  | UT Net *vs*  UT Flight/Cone | 16.41  (15.76, 17.08); <.0001 * | 17.44  (15.60, 19.49); <.0001 * | 6.25  (5.92, 6.59); <.0001 * | 7.25  (6.95, 7.56); <.0001 * |

**Table S5. Treatment-Location** **comparisons within *An. gambiae*** **strain (Kisumu, N’gousso, Banfora and VK7) and host (present or absent).** A Beta-binomial Distribution model Results**.** Multiple pairwise comparisons 95% Confidence Intervals and P-values corrected using the Bonferroni adjustment. Where IS = insecticide susceptible, IR = insecticide resistant, UT = Untreated net, OS = Olyset net, P2 = PermaNet 2.0 net and * = Significant at 5% significance level.

| ***An. gambiae* strain** | **Net** | **Host absent** | | |  | **Host present** | | |
| --- | --- | --- | --- | --- | --- | --- | --- | --- |
|  |  | **n** | **KD**  **(%)** | **24h mortality (%)** |  | **n** | **KD**  **(%)** | **24h mortality (%)** |
| **Kisumu** | P2 | 105 | 100 | 100 |  | 110 | 100 | 100 |
|  | OS | 105 | 99 | 98 |  | 100 | 100 | 100 |
| **N'gousso** | P2 | 120 | 100 | 100 |  | 113 | 100 | 100 |
|  | OS | 100 | 99 | 98 |  | 90 | 98 | 98 |
| **Banfora** | P2 | 109 | 98 | 50 |  | 135 | 91 | 50 |
|  | OS | 112 | 69 | 50 |  | 123 | 54 | 40 |
| **VK7** | P2 | 113 | 77 | 12 |  | 119 | 48 | 14 |
|  | OS | 107 | 5 | 5 |  | 113 | 42 | 4 |

**Table S6. Knock-down (KD) (1h) and mortality (24h)** after net exposure for insecticide susceptible (Kisumu and N’gousso) and insecticide resistant (VK7 and Banfora) strains of *An. gambiae s.l.* against PermaNet 2.0 (P2) and Olyset (OS) ITNs, in VCT validation experiments.

|  | | | | **Frequency (%)** | | |  | **Odds Ratio (95% Confidence Interval); P-value** | |
| --- | --- | --- | --- | --- | --- | --- | --- | --- | --- |
| **Host** | **Strain (IR)** | **Time (hrs)** | **Fed** | **UT** | **P2** | **OS** |  | **P2 vs UT** | **OS vs UT** |
| **Absent** | Banfora | 1 | Yes | 84 (85.71) | 0 (0.00) | 11 (10.68) |  | - | 0.03  (0.01, 0.10); <.0001 * |
|  |  |  | No | 14 (14.29) | 74 (100.00) | 92 (89.32) |  |  |  |
|  |  | 24 | Yes | 11 (78.57) | 42 (56.76) | 19 (20.65) |  | 0.36  (0.10, 1.32); 0.1246 | 0.07  (0.02, 0.35); 0.0009 * |
|  |  |  | No | 3 (21.43) | 32 (43.24) | 73 (79.35) |  |  |  |
|  | VK7 | 1 | Yes | 104 (86.67) | 13 (12.15) | 84 (79.25) |  | 0.02  (0.01, 0.05); <.0001 * | 0.55  (0.28, 1.07); 0.0794 |
|  |  |  | No | 16 (13.33) | 94 (87.85) | 22 (20.75) |  |  |  |
|  |  | 24 | Yes | 2 (12.50) | 77 (81.91) | 15 (68.18) |  | 25.54  (5.46, 119.50); <.0001 * | 12.06  (2.10, 69.14); 0.0052 * |
|  |  |  | No | 14 (87.50) | 17 (18.09) | 7 (31.82) |  |  |  |
| **Present** | Banfora | 1 | Yes | 106 (93.81) | 9 (10.34) | 37 (44.05) |  | 0.01  (0.00, 0.03); <.0001 * | 0.05  (0.02, 0.14); <.0001 * |
|  |  |  | No | 7 (6.19) | 78 (89.66) | 47 (55.95) |  |  |  |
|  |  | 24 | Yes | 5 (71.43) | 38 (48.72) | 16 (34.04) |  | 0.83  (0.12, 5.73); 0.8458 | 0.30  (0.05, 1.89); 0.1998 |
|  |  |  | No | 2 (28.57) | 40 (51.28) | 31 (65.96) |  |  |  |
|  | VK7 | 1 | Yes | 115 (93.50) | 19 (18.45) | 85 (77.27) |  | 0.01  (0.00, 0.04); <.0001 * | 0.11  (0.03, 0.39); 0.0006 * |
|  |  |  | No | 8 (6.50) | 84 (81.55) | 25 (22.73) |  |  |  |
|  |  | 24 | Yes | 7 (87.50) | 65 (77.38) | 18 (72.00) |  | 0.42  (0.08, 2.14); 0.2946 | 0.22  (0.03, 1.39); 0.1064 |
|  |  |  | No | 1 (12.50) | 19 (22.62) | 7 (28.00) |  |  |  |

**Table S7. Treatment comparisons** - **Willingness to refeed at 1 or 24 hours within *An. gambiae* strain and host (present or absent)**. A Binary Logistic Regression model fitted using Generalised Estimating Equation. Where IR = insecticide resistant, UT = Untreated net, OS = Olyset net, P2 = PermaNet 2.0 net and * = Significant at 5% significance level.

|  | | | | **Frequency (%)** | |  | **Odds Ratio**  **(95% Confidence Interval); P-value** | |
| --- | --- | --- | --- | --- | --- | --- | --- | --- |
| **Strain (IR)** | **Treatment** | **Time (hrs)** | **Fed** | **Present** | **Absent** |  | | **Present vs Absent** |
| **Banfora** | UT | 1 | Yes | 106 (93.81) | 84 (85.71) |  | | 11.38  (1.70, 76.36); 0.0123 * |
|  |  |  | No | 7 (6.19) | 14 (14.29) |  | |  |
|  |  | 24 | Yes | 5 (71.43) | 11 (78.57) |  | | 0.24  (0.00, 13.88); 0.4929 |
|  |  |  | No | 2 (28.57) | 3 (21.43) |  | |  |
|  | P2 | 1 | Yes | 9 (10.34) | 0 (0.00) |  | | - |
|  |  |  | No | 78 (89.66) | 74 (100.00) |  | |  |
|  |  | 24 | Yes | 38 (48.72) | 42 (56.76) |  | | 0.71  (0.24, 2.05); 0.5217 |
|  |  |  | No | 40 (51.28) | 32 (43.24) |  | |  |
|  | OS | 1 | Yes | 37 (44.05) | 11 (10.68) |  | | 7.20  (2.88, 17.97); <.0001 * |
|  |  |  | No | 47 (55.95) | 92 (89.32) |  | |  |
|  |  | 24 | Yes | 16 (34.04) | 19 (20.65) |  | | 1.86  (0.62, 5.53); 0.2668 |
|  |  |  | No | 31 (65.96) | 73 (79.35) |  | |  |
| **VK7** | UT | 1 | Yes | 115 (93.50) | 104 (86.67) |  | | 1.85  (0.43, 7.95); 0.4097 |
|  |  |  | No | 8 (6.50) | 16 (13.33) |  | |  |
|  |  | 24 | Yes | 7 (87.50) | 2 (12.50) |  | | 15.71  (1.93, 127.62); 0.0100 * |
|  |  |  | No | 1 (12.50) | 14 (87.50) |  | |  |
|  | P2 | 1 | Yes | 19 (18.45) | 13 (12.15) |  | | 1.62  (0.60, 4.38); 0.3450 |
|  |  |  | No | 84 (81.55) | 94 (87.85) |  | |  |
|  |  | 24 | Yes | 65 (77.38) | 77 (81.91) |  | | 0.64  (0.30, 1.39); 0.2621 |
| **VK7 cont.** | P2 cont. |  | No | 19 (22.62) | 17 (18.09) |  | |  |
|  | OS | 1 | Yes | 85 (77.27) | 84 (79.25) |  | | 0.68  (0.23, 1.99); 0.4824 |
|  |  |  | No | 25 (22.73) | 22 (20.75) |  | |  |
|  |  | 24 | Yes | 18 (72.00) | 15 (68.18) |  | | 0.34  (0.05, 2.33); 0.2704 |
|  |  |  | No | 7 (28.00) | 7 (31.82) |  | |  |

**Table S8. Host comparisons** - **Willingness to refeed at 1 or 24 hours within *An. gambiae* strain and treatment**. A Binary Logistic Regression model fitted using Generalised Estimating Equation. Where IR = insecticide resistant, UT = Untreated net, OS = Olyset net, P2 = PermaNet 2.0 net and * = Significant at 5% significance level.

|  | | **N; mean µg/ml (SD)** | | |  | **Mean difference µg/ml** **(95% CI); P-value** | |
| --- | --- | --- | --- | --- | --- | --- | --- |
| **Host** | **Strain** | **UT** | **P2** | **OS** |  | **P2 vs UT** | **OS vs UT** |
| **Absent** | Banfora | 82; 15.08 (6.74) | 38; 9.15 (7.32) | 28; 7.83 (6.58) |  | -5.27  (-8.20, -2.34); 0.0004 * | -5.29  (-8.40, -2.18); 0.0009 * |
|  | VK7 | 77; 11.21 (5.15) | 73; 9.45 (6.37) | 85; 9.44 (6.48) |  | -1.86  (-4.11, 0.38); 0.1034 | -2.05  (-3.48, -0.62); 0.0049 * |
| **Present** | Banfora | 59; 10.26 (6.68) | 45; 7.83 (6.76) | 43; 7.82 (5.78) |  | -1.19  (-3.92, 1.55); 0.3946 | -1.81  (-4.24, 0.63); 0.1462 |
|  | VK7 | 89; 15.28 (6.63) | 74; 9.64 (7.22) | 93; 12.70 (6.35) |  | -2.46  (-4.90, -0.02); 0.0477 * | -0.72  (-2.68, 1.24); 0.4729 |

**Table S9. Treatment comparisons** **– Blood size volume within *An. gambiae* strain and host (present or absent)**. A Linear Regression model fitted using Generalised Estimating Equation. Where IS = insecticide susceptible, IR = insecticide resistant, UT = Untreated net, OS = Olyset net, P2 = PermaNet 2.0, CI = Confidence Interval net and * = Significant at 5% significance level.

|  | | **N; mean µg/ml** **(SD)** | |  | **Mean difference µg/ml** **(95% CI); P-value** |
| --- | --- | --- | --- | --- | --- |
| **Treatment** | **Strain** | **Present** | **Absent** |  | **Present vs Absent** |
| **UT** | Banfora | 59; 10.26 (6.68) | 82; 15.08 (6.74) |  | -1.66  (-4.37, 1.06); 0.2316 |
|  | VK7 | 89; 15.28 (6.63) | 77; 11.21 (5.15) |  | 2.86  (1.25, 4.47); 0.0005 * |
| **P2** | Banfora | 45; 7.83 (6.76) | 38; 9.15 (7.32) |  | 2.43  (-0.17, 5.03); 0.0666 |
|  | VK7 | 74; 9.64 (7.22) | 73; 9.45 (6.37) |  | 2.26  (0.06, 4.46); 0.0437 * |
| **OS** | Banfora | 43; 7.82 (5.78) | 28; 7.83 (6.58) |  | 1.82  (-1.14, 4.79); 0.2273 |
|  | VK7 | 93; 12.70 (6.35) | 85; 9.44 (6.48) |  | 4.23  (2.22, 6.24); <.0001 * |

**Table S10. Host comparisons** **– Blood meal size within *An. gambiae* strain and treatment**. A Linear Regression model fitted using Generalised Estimating Equation. Where IS = insecticide susceptible, IR = insecticide resistant, UT = Untreated net, OS = Olyset net, P2 = PermaNet 2.0 net and * = Significant at 5% significance level.

|  | | | **Model without haematin** | | | |  | **Model with haematin** | | | | | |
| --- | --- | --- | --- | --- | --- | --- | --- | --- | --- | --- | --- | --- | --- |
| **Host** | **Strain (IR)** | **Statistics** | **UT** | **P2** | **OS** | **All** |  | **UT** | | **P2** | | **OS** | **All** |
| **Absent** | BF | n (%) | 91(30.74) | 102(34.46) | 103 (34.80) | 296 |  | 82 (55.41) | 38 (25.68) | | 28 (18.92) | | 148 |
|  |  | Mean (SD) | 14.43(7.15) | 6.61(8.19) | 5.74 (7.85) | 8.71 (8.63) |  | 15.72 (6.30) | 15.34 (7.48) | | 16.43 (7.80) | | 15.76 (6.88) |
|  |  | Min-Max | 1.00-35.00 | 1.00-33.00 | 1.00-32.00 | 1.00-35.00 |  | 4.00-35.00 | 6.00-33.00 | | 4.00-32.00 | | 4.00-35.00 |
|  |  | Median  (95%CI) | 14.00  (12.00 - 15.00) | 1.00  (1.00 - 4.00) | 1.00  (1.00 - 2.00) | 5.00  (3.00 - 9.00) |  | 14.50  (13.00 - 16.00) | 13.50  (12.00 - 14.00) | | 15.00  (12.00 - 19.00) | | 14.00  (13.00 - 15.00) |
|  |  | Yes n/total  (%) * | 20/91  (21.98) | 72/102  (70.59) | 79/103  (76.70) | 171/296  (57.77) |  | 11/82  (13.41) | 8/38  (21.05) | | 5/28  (17.86) | | 24/148  (16.22) |
|  |  | Mean (SD) * | 5.10(2.77) | 2.11(2.24) | 1.87(1.73) | 2.35(2.31) |  | 7.09(2.07) | 7.63(1.06) | | 5.80(1.92) | | 7.00(1.82) |
|  |  | Median  (95%CI) * | 4.00  (3.00 - 7.00) | 1.00 | 1.00 | 1.00 |  | 7.00  (4.00 - 9.00) | 7.50  (6.00 - 9.00) | | 5.00  (4.00 - 9.00) | | 7.00  (6.00 - 9.00) |
|  | VK | n (%) | 100 (33.56) | 98(32.89) | 100(33.56) | 298 |  | 73(31.60) | 73(31.60) | | 85(36.80) | | 231 |
|  |  | Mean (SD) | 7.86(6.51) | 11.27(7.93) | 9.65(5.68) | 9.58(6.88) |  | 9.48(6.19) | 14.01(6.83) | | 10.81(5.27) | | 11.40(6.34) |
|  |  | Min-Max | 1.00-31.00 | 1.00-37.00 | 1.00-25.00 | 1.00-37.00 |  | 4.00-31.00 | 4.00-37.00 | | 4.00-25.00 | | 4.00-37.00 |
|  |  | Median (95%CI) | 5.00 | 10.00  (6.00 - 14.00) | 8.00  (6.00 - 12.00) | 7.00  (6.00 - 9.00) |  | 6.00  (5.00 - 10.00) | 15.00  (12.00 - 17.00) | | 12.00  (8.00 - 12.00) | | 11.00  (8.00 - 12.00) |
|  |  | Yes n/total (%) * | 69/100  (69.00) | 49/98  (50.00) | 52/100  (52.00) | 170/298  (57.05) |  | 43/73  (58.90) | 25/73  (34.25) | | 38/85  (44.71) | | 106/231  (45.89) |
|  |  | Mean (SD) * | 11.19(7.71) | 9.69(5.96) | 8.19(6.65) | 9.73(6.92) |  | 11.27(7.93) | 9.65(5.68) | | 7.86(6.51) | | 9.58(6.88) |
|  |  | Median  (95%CI) * | 5.00  (4.00 - 5.00) | 5.00  (3.00 - 5.00) | 5.00 | 5.00  (4.00 - 5.00) |  | 5.00 | 6.00  (5.00 - 7.00) | | 5.00  (5.00 - 6.00) | | 5.00 |
| **Present** | BF | n (%) | 67(23.02) | 104(35.74) | 120(41.24) | 291 |  | 59(40.14) | 45(30.61) | | 43(29.25) | | 147 |
|  |  | Mean (SD) | 13.67(7.05) | 7.80(8.63) | 6.28(7.82) | 8.52(8.44) |  | 14.90(6.46) | 15.87(7.36) | | 14.07(7.60) | | 14.95(7.07) |
|  |  | Min-Max | 1.00-42.00 | 1.00-37.00 | 1.00-32.00 | 1.00-42.00 |  | 5.00-42.00 | 6.00-37.00 | | 4.00-32.00 | | 4.00-42.00 |
|  |  | Median  (95%CI) | 13.00  (12.00 - 15.00) | 5.00  (1.00 - 6.00) | 1.00  (1.00 - 4.00) | 5.00  (5.00 - 7.00) |  | 14.00  (13.00 - 16.00) | 14.00  (12.00 - 17.00) | | 13.00  (11.00 - 16.00) | | 14.00  (13.00 - 15.00) |
|  |  | Yes n/total  (%) * | 20/67  (29.85) | 67/104  (64.42) | 87/120  (72.50) | 174/291  (59.79) |  | 13/59  (22.03) | 8/45  (17.78) | | 12/43  (27.91) | | 33/147  (22.45) |
|  |  | Mean (SD) * | 5.90(2.49) | 2.25(2.16) | 2.02(1.84) | 2.56(2.37) |  | 7.31(1.55) | 6.75(0.71) | | 5.42(1.44) | | 6.48(1.56) |
|  |  | Median  (95%CI) * | 6.00  (4.00 - 8.00) | 1.00 | 1.00 | 1.00 |  | 7.00  (5.00 - 9.00) | 7.00  (6.00 - 7.00) | | 5.00  (4.00 - 6.00) | | 7.00  (5.00 - 7.00) |
|  | VK | n (%) | 108(33.75) | 107(33.44) | 105(32.81) | 320 |  | 89(34.90) | 73(28.63) | | 93(36.47) | | 255 |
|  |  | Mean (SD) | 12.18(6.70) | 11.27(7.72) | 15.06(7.63) | 12.82(7.51) |  | 13.85(6.04) | 14.84(5.80) | | 16.32(6.92) | | 15.04(6.37) |
|  |  | Min-Max | 1.00-39.00 | 1.00-33.00 | 1.00-38.00 | 1.00-39.00 |  | 4.00-39.00 | 6.00-33.00 | | 6.00-38.00 | | 4.00-39.00 |
|  |  | Median  (95%CI) | 12.00  (11.00 - 14.00) | 12.00  (10.00 - 14.00) | 15.00  (12.00 - 17.00) | 13.00  (12.00 - 14.00) |  | 14.00  (12.00 - 15.00) | 15.00  (12.00 - 15.00) | | 16.00  (13.00 - 18.00) | | 15.00  (14.00 - 15.00) |
|  |  | Yes n/total  (%) * | 36/108  (33.33) | 42/107  (39.25) | 23/105  (21.90) | 101/320  (31.56) |  | 18/89  (20.22) | 12/73  (16.44) | | 13/93  (13.98) | | 43/255  (16.86) |
|  |  | Mean (SD) * | 4.78(1.76) | 3.36(2.76) | 5.26(2.53) | 4.30(2.50) |  | 5.89(1.53) | 7.33(0.89) | | 7.00(1.15) | | 6.63(1.40) |
|  |  | Median  (95%CI) * | 4.00  (4.00 - 5.00) | 2.00  (1.00 - 4.00) | 6.00  (4.00 - 6.00) | 4.00  (4.00 - 5.00) |  | 6.00  (5.00 - 6.00) | 7.00  (6.00 - 8.00) | | 7.00  (6.00 - 8.00) | | 6.00  (6.00 - 7.00) |

**Table S11.** **Descriptive statistics for longevity data within host, *An. gambiae* strain and net treatment**. Where BF = Banfora, VK= VK7, UT = Untreated net, P2 = PermaNet 2.0 net, OS = Olyset net, IR = insecticide resistant, n = sample size, % = percentage, SD = Standard Deviation, Min = Minimum, Max = Maximum, CI = Confidence Interval, Yes n = number of mosquitoes that died within 9 days and * results for mosquitoes that died within 9 days post-exposure only.

|  |  |  | **Hazard Ratio (95% Confidence Interval); *P*-value** | |
| --- | --- | --- | --- | --- |
| **Host** | **Strain (IR)** | **Variable** | **Model without haematin** | **Model with haematin** |
| **Absent** | Banfora | Treatment: UT (Ref) |  |  |
|  |  | Treatment: P2 | 2.03  (1.40, 2.93); 0.0002 * | 2.00  (0.28, 14.34); 0.4903 |
|  |  | Treatment: OS | 1.66  (1.09, 2.52); 0.0189 * | 0.71  (0.01, 39.39); 0.8667 |
|  |  | Time fed: 1 hr (Ref) |  |  |
|  |  | Time fed: 24 hrs | 0.79  (0.36, 1.70); 0.5417 | 0.36  (0.03, 5.16); 0.4527 |
|  |  | Time fed: Unfed | 25.65  (12.38, 53.16); <0.0001 * | - |
|  |  | Time fed: Other | 31.80  (15.80, 63.99); <0.0001 * | 0.00  (0.00, 9.54); 0.8993 |
|  |  | Net proportion | 0.88  (0.59, 1.31); 0.5225 | 0.04  (0.00, 4.49); 0.1788 |
|  |  | Wingspan | 0.66  (0.36, 1.23); 0.1946 | 0.26  (0.01, 6.25); 0.4084 |
|  |  | Haematin | - | 0.87  (0.77, 0.98); 0.0198 * |
|  | VK7 | Treatment: UT (Ref) |  |  |
|  |  | Treatment: P2 | 0.55  (0.32 -0.97); 0.0373* | 0.30  (0.17 -0.55); 0.0001* |
|  |  | Treatment: OS | 0.63  (0.42, 0.94); 0.0222 * | 0.48  (0.29, 0.81); 0.0057 * |
|  |  | Time fed: 1 hr (Ref) |  |  |
|  |  | Time fed: 24 hrs | 0.78  (0.45, 1.34); 0.3605 | 1.00  (0.65, 1.56); 0.9923 |
|  |  | Time fed: Unfed | 6.75  (3.53, 12.89); <0.0001 * | - |
|  |  | Time fed: Other | 13.68  (7.17, 26.10); <0.0001 * | - |
|  |  | Net proportion | 0.81  (0.33, 2.04); 0.6613 | 0.84  (0.25, 2.80); 0.7758 |
|  |  | Wingspan | 1.67  (0.67, 4.13); 0.2714 | 2.24  (0.73, 6.90); 0.1580 |
|  |  | Haematin | - | 0.93  (0.90, 0.97); 0.0008 * |
| **Present** | Banfora | Treatment: UT (Ref) |  |  |
|  |  | Treatment: P2 | 2.01  (1.00, 4.06); 0.0510 | 0.51  (0.17, 1.55); 0.2362 |
|  |  | Treatment: OS | 2.83  (1.36, 5.87); 0.0053 * | 1.45  (0.53, 3.99); 0.4745 |
|  |  | Time fed: 1 hr (Ref) |  |  |
|  |  | Time fed: 24 hrs | 0.67  (0.31, 1.44); 0.3076 | 2.84  (1.08, 7.50); 0.0347 * |
| **Present cont.** | Banfora cont. | Time fed: Unfed | 2.32  (1.16, 4.62); 0.0167 * | 0.65  (0.14, 2.97); 0.5762 |
|  |  | Time fed: Other | 9.38  (4.13, 21.29); <0.0001 * | - |
|  |  | Net proportion | 1.83  (0.87, 3.85); 0.1117 | 2.78  (0.23, 34.07); 0.4236 |
|  |  | Wingspan | 0.69  (0.43, 1.12); 0.1354 | 3.61  (0.21, 61.27); 0.3747 |
|  |  | Haematin | - | 0.90  (0.83, 0.97); 0.0063 * |
|  | VK7 | Treatment: UT (Ref) |  |  |
|  |  | Treatment: P2 | 0.93  (0.49, 1.78); 0.8333 | 0.47  (0.18, 1.27); 0.1390 |
|  |  | Treatment: OS | 0.35  (0.16, 0.74); 0.0058 * | 0.50  (0.18, 1.40); 0.1895 |
|  |  | Time fed: 1 hr (Ref) |  |  |
|  |  | Time fed: 24 hrs | 0.63  (0.35, 1.13); 0.1208 | 0.52  (0.21, 1.30); 0.1622 |
|  |  | Time fed: Unfed | 1.89  (0.99, 3.62); 0.0555 | 1.08  (0.37, 3.16); 0.8902 |
|  |  | Time fed: Other | 22.44  (7.26, 69.37); <0.0001 * | 0.00  (0.00, 0.02); 0.0274 * |
|  |  | Net proportion | 0.47  (0.06, 3.52); 0.4602 | 1.08  (0.05, 22.25); 0.9618 |
|  |  | Wingspan | 0.69  (0.22, 2.18); 0.5233 | 2.42  (0.43, 13.74); 0.3195 |
|  |  | Haematin | - | 0.91  (0.85, 0.97); 0.0072 * |

**Table S12. Net treatment comparisons** - **Mortality within 9 days** **within *An. gambiae* strain and host**. A Weighted Cox Regression Model fitted adjusted for other predictors (time fed, wingspan, haematin and net proportion). Where UT = Untreated net, P2 = PermaNet 2.0 net, OS = Olyset net, IR = insecticide resistant, Ref = Reference group, Net proportion = Mean proportion of mosquitoes on the net during exposure (behaviour data) and * = Significant at 5% significance level. Censoring at 9 days.

|  |  |  | **Hazard Ratio (95% Confidence Interval); *P*-value** | |
| --- | --- | --- | --- | --- |
| **Treatment** | **Strain (IR)** | **Variable** | **Model without haematin** | **Model with haematin** |
| **UT** | Banfora | Host: Absent (Ref) |  |  |
|  |  | Host: Present | 1.34  (0.43, 4.18); 0.6125 | 1.06  (0.18, 6.24); 0.9479 |
|  |  | Time fed: 1 hr (Ref) |  |  |
|  |  | Time fed: 24 hrs | 1.61  (0.43, 6.07); 0.4794 | 1.28  (0.22, 7.52); 0.7858 |
|  |  | Time fed: Unfed | 7.62  (1.76, 32.93); 0.0066 * | 0.95  (0.14, 6.23); 0.9537 |
|  |  | Time fed: Other | 64.98  (16.97, 248.88); <0.0001 * | - |
|  |  | Net proportion | 0.39  (0.02, 5.99); 0.4965 | 0.66  (0.01, 33.53); 0.8370 |
|  |  | Wingspan | 0.11  (0.01, 0.81); 0.0306 * | 0.97  (0.05, 17.14); 0.9809 |
|  |  | Haematin | - | 0.84  (0.75, 0.94); 0.0025 * |
|  | VK7 | Host: Absent (Ref) |  |  |
|  |  | Host: Present | 0.51  (0.30, 0.86); 0.0108 * | 0.36  (0.12, 1.07); 0.0655 |
|  |  | Time fed: 1 hr (Ref) |  |  |
|  |  | Time fed: 24 hrs | 0.60  (0.21, 1.68); 0.3297 | 0.36  (0.12, 1.11); 0.0752 |
|  |  | Time fed: Unfed | 4.65  (2.41, 8.98); <0.0001 * | - |
|  |  | Time fed: Other | 41.46  (24.40, 70.45); <0.0001 * | - |
|  |  | Net proportion | 2.52  (0.21, 30.72); 0.4685 | 1.22  (0.02, 73.45); 0.9233 |
|  |  | Wingspan | 0.54  (0.14, 2.09); 0.3737 | 0.55  (0.09, 3.47); 0.5268 |
|  |  | Haematin | - | 0.90  (0.86, 0.94); <0.0001 * |
| **P2** | Banfora | Host: Absent (Ref) |  |  |
|  |  | Host: Present | 0.64  (0.49, 0.83); 0.0010 * | 1.12  (0.32, 3.87); 0.8587 |
|  |  | Time fed: 1 hr (Ref) |  |  |
|  |  | Time fed: 24 hrs | 0.27  (0.10, 0.79); 0.0171 * | 0.57  (0.11, 2.82); 0.4903 |
|  |  | Time fed: Unfed | 3.68  (1.33, 10.19); 0.0121 * | 0.38  (0.09, 1.69); 0.2044 |
|  |  | Time fed: Other | 10.83  (3.88, 30.18); <0.0001 * | - |
|  |  | Net proportion | 0.94  (0.27, 3.28); 0.9216 | 4.70  (0.06, 352.57); 0.4823 |
|  |  | Wingspan | 0.77  (0.46, 1.30); 0.3286 | 0.21  (0.01, 4.47); 0.3149 |
|  |  | Haematin | - | 0.95  (0.87, 1.04); 0.2803 |
|  | VK7 | Host: Absent (Ref) |  |  |
|  |  | Host: Present | 0.65  (0.40, 1.06); 0.0836 | 0.34  (0.14, 0.86); 0.0231 * |
|  |  | Time fed: 1 hr (Ref) |  |  |
|  |  | Time fed: 24 hrs | 1.31  (0.60, 2.82); 0.4973 | 1.58  (0.64, 3.89); 0.3162 |
|  |  | Time fed: Unfed | 5.56  (2.41, 12.81); 0.0001 * | 1.86  (0.57, 6.08); 0.3054 |
|  |  | Time fed: Other | 26.72  (12.86, 55.51); <0.0001 * | - |
|  |  | Net proportion | 1.82  (0.66, 5.01); 0.2485 | 4.26  (0.96, 18.86); 0.0564 |
|  |  | Wingspan | 1.93  (0.66, 5.68); 0.2327 | 2.54  (0.31, 21.02); 0.3865 |
|  |  | Haematin | - | 0.89  (0.82, 0.96); 0.0038 * |
| **OS** | Banfora | Host: Absent (Ref) |  |  |
|  |  | Host: Present | 0.91  (0.68, 1.21); 0.4985 | 3.21  (0.63, 16.33); 0.1608 |
|  |  | Time fed: 1 hr (Ref) |  |  |
|  |  | Time fed: 24 hrs | 0.47  (0.19, 1.15); 0.0993 | 1.83  (0.80, 4.15); 0.1497 |
|  |  | Time fed: Unfed | 4.57  (2.27, 9.17); <0.0001 * | 0.32  (0.03, 3.29); 0.3378 |
|  |  | Time fed: Other | 7.12  (3.32, 15.27); <0.0001 * | 0.00  (0.00, 7.59); 0.3780 |
|  |  | Net proportion | 1.40  (0.88, 2.22); 0.1534 | 0.57  (0.00, 72.59); 0.8205 |
|  |  | Wingspan | 0.59  (0.33, 1.07); 0.0835 | 25.90  (0.10, 6408.23); 0.2471 |
|  |  | Haematin | - | 0.92  (0.84, 1.02); 0.1079 |
|  | VK7 | Host: Absent (Ref) |  |  |
|  |  | Host: Present | 0.09  (0.04, 0.22); <0.0001 * | 0.08  (0.03, 0.21); <0.0001 * |
|  |  | Time fed: 1 hr (Ref) |  |  |
|  |  | Time fed: 24 hrs | 0.91  (0.54, 1.52); 0.7222 | 1.03  (0.58, 1.83); 0.9155 |
|  |  | Time fed: Unfed | 5.92  (2.34, 14.96); 0.0002 * | 3.96  (0.58, 27.20); 0.1622 |
|  |  | Time fed: Other | 6.78  (2.29, 20.07); 0.0005 * | 0.00  (0.00, 5314.96); 0.0966 |
|  |  | Net proportion | 0.03  (0.00, 0.23); 0.0006 * | 0.05  (0.01, 0.32); 0.0018 * |
|  |  | Wingspan | 0.99  (0.24, 4.01); 0.9863 | 2.54  (0.48, 13.46); 0.2718 |
|  |  | Haematin | - | 0.98  (0.93, 1.04); 0.5754 |

**Table S13. Host comparisons - Mortality within 9 days** **within *An. gambiae* strain and net treatment.** A Weighted Cox Regression Model fitted adjusted for other predictors (time fed, wingspan, haematin and net proportion). Where UT = Untreated net, P2 = PermaNet 2.0 net, OS = Olyset net, IR = insecticide resistant, Ref = Reference group, Net proportion = Mean proportion of mosquitoes on the net during exposure (behaviour data) and * = Significant at 5% significance level. Censoring at 9 days.
